# The regional diversity of gut microbiome along the GI tract of male C57BL/6 mice

**DOI:** 10.1186/s12866-021-02099-0

**Published:** 2021-02-12

**Authors:** Enkhchimeg Lkhagva, Hea-Jong Chung, Jinny Hong, Wai Hong Wilson Tang, Sang-Il Lee, Seong-Tshool Hong, Seungkoo Lee

**Affiliations:** 1 Department of Biomedical Sciences and Institute for Medical Science, Chonbuk National University Medical School, Jeonju, South Korea; 2 Gwangju Center, Korea Basic Science Institute, Gwangju, South Korea; 3 Department of Biochemistry, Case Western Reserve University, Cleveland, OH USA; 4 Department of Cardiovascular Medicine, Heart and Vascular Institute, Cleveland, OH USA; 5 Division of Rheumatology, Gyeongsang National University Hospital, Jinju, South Korea; 6 Department of Anatomic Pathology, School of Medicine, Kangwon National University, Kangwon National University Hospital, 1 Gangwondaehak-gil, Chuncheon, Gangwon 24341 South Korea

**Keywords:** Gut microbiome, α- diversity, β-Diversity

## Abstract

**Background:**

The proliferation and survival of microbial organisms including intestinal microbes are determined by their surrounding environments. Contrary to popular myth, the nutritional and chemical compositions, water contents, O2 contents, temperatures, and pH in the gastrointestinal (GI) tract of a human are very different in a location-specific manner, implying heterogeneity of the microbial composition in a location-specific manner.

**Results:**

We first investigated the environmental conditions at 6 different locations along the GI tract and feces of ten weeks’ old male SPF C57BL/6 mice. As previously known, the pH and water contents of the GI contents at the different locations of the GI tract were very different from each other in a location-specific manner, and none of which were not even similar to those of feces. After confirming the heterogeneous nature of the GI contents in specific locations and feces, we thoroughly analyzed the composition of the microbiome of the GI contents and feces. 16S rDNA-based metagenome sequencing on the GI contents and feces showed the presence of 13 different phyla. The abundance of Firmicutes gradually decreased from the stomach to feces while the abundance of Bacteroidetes gradually increased. The taxonomic α-diversities measured by ACE (Abundance-based Coverage Estimator) richness, Shannon diversity, and Fisher’s alpha all indicated that the diversities of gut microbiome at colon and cecum were much higher than that of feces. The diversities of microbiome compositions were lowest in jejunum and ileum while highest in cecum and colon. Interestingly, the diversities of the fecal microbiome were lower than those of the cecum and colon. Beta diversity analyses by NMDS plots, PCA, and unsupervised hierarchical clustering all showed that the microbiome compositions were very diverse in a location-specific manner. Direct comparison of the fecal microbiome with the microbiome of the whole GI tracts by α-and β-diversities showed that the fecal microbiome did not represent the microbiome of the whole GI tract.

**Conclusion:**

The fecal microbiome is different from the whole microbiome of the GI tract, contrary to a baseline assumption of contemporary microbiome research work.

**Supplementary Information:**

The online version contains supplementary material available at 10.1186/s12866-021-02099-0.

## Background

One hundred trillion of microbes are resided in a typical in the intestine of human as gut microbiome whose collective genome contains 100 times more genes than our own genome [1–3]. The results of the interactions between gut microbiome and its host are various; negligible, negative, or positive [4]. Despite negative consequences in some cases, the presence of gut microbiome is essential to our health and well-being in most cases [4]. Considering the number of genes in human gut microbiome, it is not suprizing to note that the gut microbiome contributes significantly to the traits of humans as much as our genes, especially in the case of atherosclerosis, hypertension, obesity, diabetes, metabolic syndrome and its related diseases, inflammatory bowel disease (IBD), gastrointestinal tract malignancies, hepatic encephalopathy, allergies, behavior, autism, and neurological diseases [4, 5]. Alteration of the composition of the gut microbiome even affects the behavior, intelligence, mood, autism, psychology, and migraines of its host through the gut-brain axis [6]. It is now clear that the relationship between gut microbiome and humans is not merely commensal but rather a mutualistic relationship [6–9]. Thus, recent advances in gut microbiome are not only elucidating our understanding of human biology but also present a new paradigm of opportunities for development of new concepts of therapeutic agents.

Gut microbiome comprises all intestinal microorganisms residing along with the gastrointestinal (GI) tract which include commensal, symbiotic, and pathogenic microorganisms. Almost all of the current research on gut microbiome strictly rely on the metagenome sequencing analyses of the microbiome isolated from fecal samples under the baseline assumption that fecal microbiome represents the whole gut microbiome or at least similar [10, 11]. The GI tract is a hollow organ system but divided into sections that digests food, extracts and absorbs nutrients, and discharges waste materials in a location-specific manner. The environmental conditions such as pHs, water contents, chemical profiles, O_2_ contents, etc. in the GI tract are constantly changed location by location as the specific components of foods are mechanically and enzymatically broken down into substances for absorption into the bloodstream [12].

The growth of microbial organisms is ultimately determined by environmental factors such as chemical components, water contents, O_2_ contents, temperatures, and pH [13–15]. Intestinal microbial organisms are not an exception. The nutritional and chemical compositions, water contents, O_2_ contents, temperatures, and pH in the gastrointestinal (GI) tract of human are very different in a location-specific manner [16–20], which implies that the compositions of gut microbiome in the GI tract could also differ in a location-specific manner. Since none of these environmental factors in feces are represented in any part of the GI tract even in the large intestine, it would be reasonable to question whether the fecal microbiome does represent the microbiome of the gastrointestinal tract or not.

Considering these variances in the GI tract, we investigated variations of gut microbiome at different locations of the GI tract, following comparison of the compositions of the microbiome in the GI tract with the fecal microbiome by using thorough statistical methods. This work showed that the compositions of gut microbiome were constantly changing at a location-specific manner reflecting its environmental difference.

## Results

### The environmental conditions in the GI tract varied in a location-specific manner

The realization of the variable nature of environmental factors in the GI tract prompted us to investigate the possibility of the location-specific environmental variations in the GI tract by using male SPF C57BL/6 mice. The whole GI tracts of mice of ten weeks’ old were divided into six parts (Figure S1), and the GI contents from each location as well as feces were collected and analyzed. As expected, the pHs and water contents of the GI contents were very different from each other in a location-specific manner along the GI tract and those of feces were not similar to the GI contents at any location (Table S1, S2), indicating heterogeneous environments along the GI tract. These results clearly showed that the environmental conditions in the GI tract vary reflecting the local function in the GI tract. The environmental condition of feces was not similar to those of any part of the GI contents, nor the overall GI content.

### Metagenome sequencing unveiled the location-specific diversity of gut microbiome in the GI tract

**Fig. 1:**
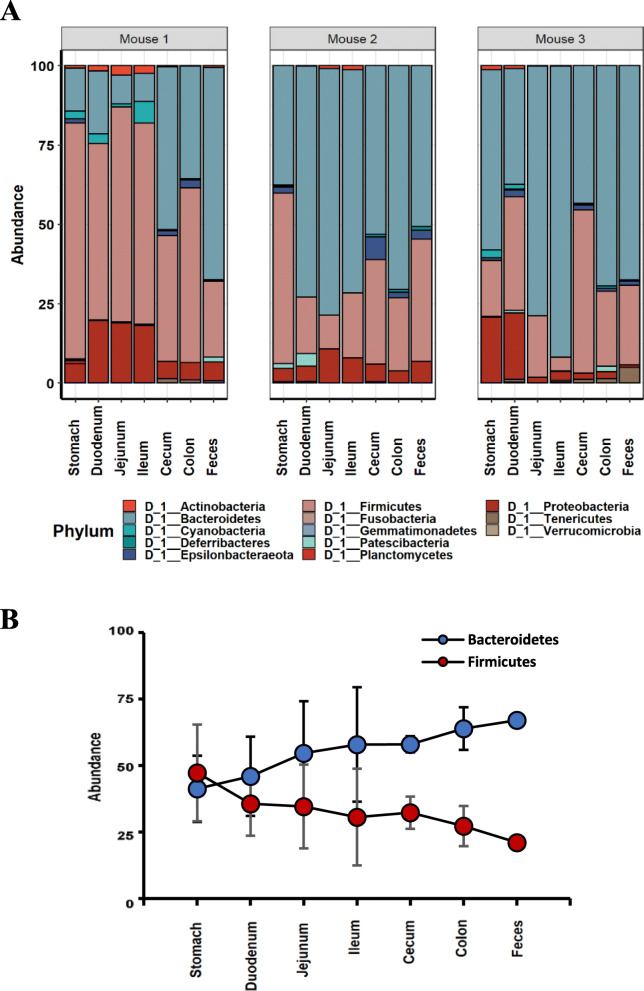
Comparison of microbial diversity at the different locations of the GI tract in the same mouse. **a**. Relative abundance of phyla occupying in the GI tract and feces. **b**. The relative abundance of Bacteroidetes and Firmicutes along the GI tract

We next investigated the diversity of gut microbiome at different locations within the same mouse by 16S rDNA-based metagenome analyses. The V3-V4 sites of the 16S rRNA genes of the isolated genomic DNAs of the gut microbiome of the GI contents were sequenced using the MiSeq™ platform (Illumina). The sequence reads containing incorrect primer, barcode sequences, sequences with more than one ambiguous base, low-quality sequences or chimeras were 2.2%, and these sequence reads were removed. The filtered 16S rDNA sequences were used to identify individual microbes by matching the 16S rDNA sequences with the SILVA reference (region V3-V4) database (https://www.arb-silva.de/). All of the identified 16S rDNA sequences were able to be classified into 13 different phyla; Bacteroidetes (51.5%), Firmicutes (35.88%), Proteobacteria (8.29%), Epsilonbacteraeota (1.26%), Cyanobacteria (0.94%), Actinobacteria (0.63%), Patescibacteria (0.5%), Deferribacteres (0.17%), Tenericutes (0.62%), Verrucomicrobia (0.08%), Planctomycetes (0.04%), Fusobacteria (0.03%), and Gemmatimonadetes (0.01%) (Fig. 1a). Interestingly, the abundance of the two most abundant groups of microbes was reversed from the stomach to feces along with the GI tract (Fig. 1b), suggesting that microbiota composition change reflecting the environmental change in the GI tract. The abundance of Firmicutes gradually decreased from the stomach to feces while the abundance of Bacteroidetes gradually increased (Fig. 1b).

### Alpha-diversity analysis showed that microbiomes in the different locations of the GI tract completely differed from each other

The gross microbiome analysis at the phylum level along the GI tract indicated that the microbiome was ever-changing along the GI tract reflecting their various environments. The microbiome in the GI tract was very different from the fecal microbiome (Fig. 1a, b, Figure S2 ~ S4), and the discrepancy depending on locations became more evident at lower taxonomic levels (Figure S5). Also, interestingly enough, the microbiome in the upper GI tracts and small intestines completely differed from those of the lower GI tracts within the same mouse and the degree of differences gradually decreased from the stomach to feces (Figure S5, Table S3 ~ S7). It should be noted that the microbiome differences of large intestines among different mice were significantly decreased, demonstrating quite similar microbiome compositions of large intestine and feces among different mice. The microbiome analysis at the class level demonstrated that Bacteroidia was unanimously abundant along the GI tracts while most abundance was observed with Bacilli and Clostridia in the stomach, with Bacilli and Erysipelotrichia in the small intestine, and with Clostridia in the large intestine and feces (Table S4). Likewise, the GI tract was unanimously abundant with the order of Bacteroidales followed by Lactobacillales and Clostridiales in the stomach, Lactobacillales and Erysipelotrichales in the small intestine, and Clostridiales in the large intestine and feces, respectively (Table S5). At the family levels, Muribaculaceae was unanimously abundant followed by Lactobacillaceae and Lachnospiraceae in the stomach, Lactobacillales in the small intestine, and Lachnospiraceae and Ruminococcaceae in the large intestine and feces, respectively (Table S6). At the genus levels, there was a clearly distinguished pattern along with the GI locations despite the presence of unidentified groups (Table S7). *Helicobacter* was in the stomach as well as large intestine but not in the small intestine. *Lactococcus, Dubosiella, Parasutterella,* and *Turicibacter* were specifically observed in the small intestine while *Helicobacter, Bacteroides, Alloprevotella, Odoribacter*, and *Alistipes* in the large intestine and feces (Table S7).

**Fig. 2:**
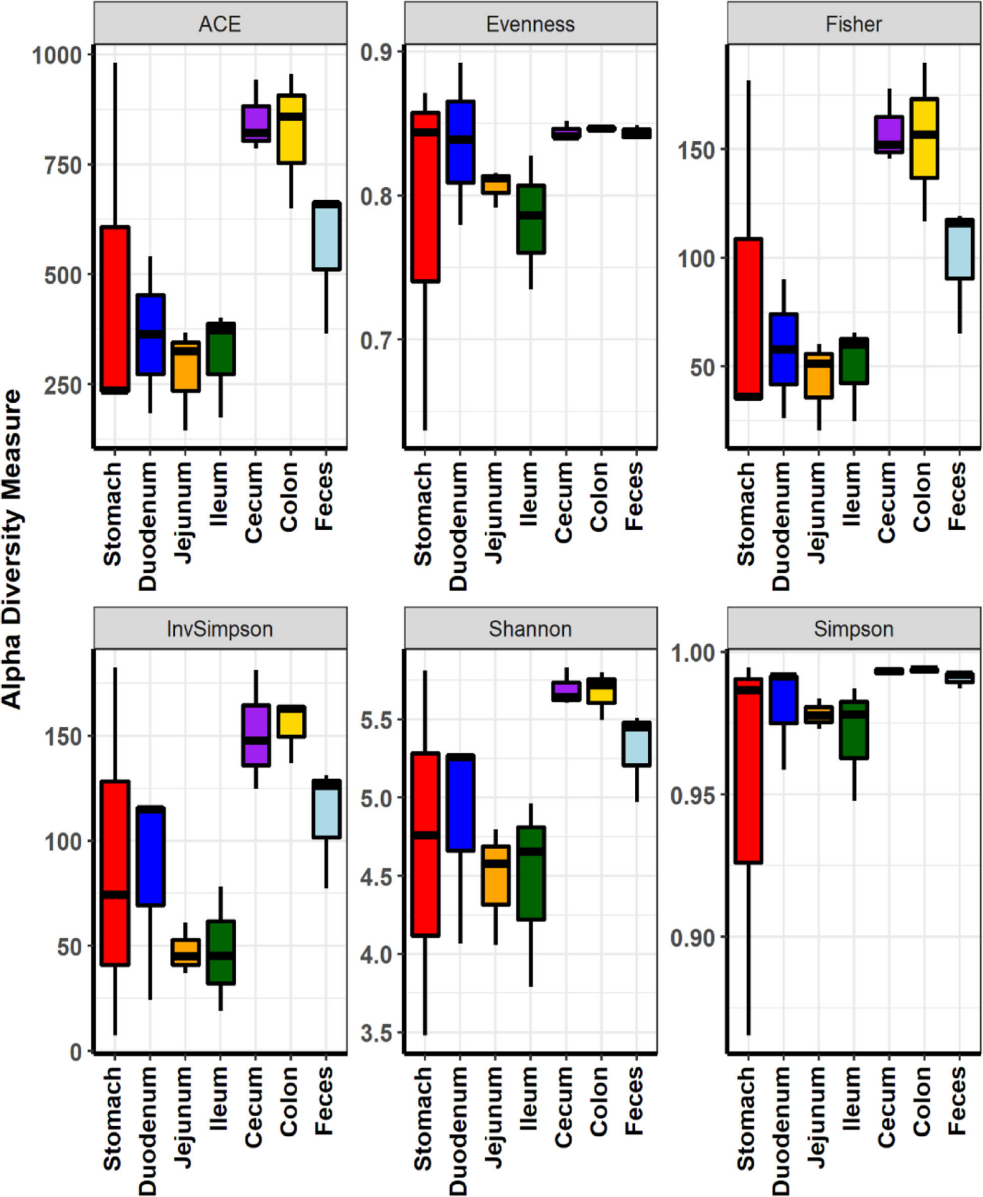
Comparison of microbial diversity at the different locations of the GI tract in the mouse by α-diversity analysis. Species richness and diversity measured by ACE richness, Evenness, Fisher’s alpha, Inverse Simpson, Shannon and Simpson diversity at the different locations of the GI tract

Our initial comparison of the microbiome compositions at locations along the GI tracts was followed by a thorough diversity analysis of the microbiome. The taxonomic α-diversities measured by ACE richness, Shannon diversity, and Fisher’s alpha all indicated that the diversities of gut microbiome at colon and cecum were much higher than that of feces (Fig. 2, Table S8). The diversities of microbiome compositions were lowest in jejunum and ileum while highest in cecum and colon. It should be noted that the diversities of the fecal microbiome were lower than those of the cecum and colon. Clearly, the α-diversity analyses indicated that the fecal microbiome did not represent the microbiome in the GI tract of its host, contrary to the general baseline assumption.

### Beta-diversity analysis confirmed that microbiomes in different locations of the GI tract completely differed from each other

**Fig. 3:**
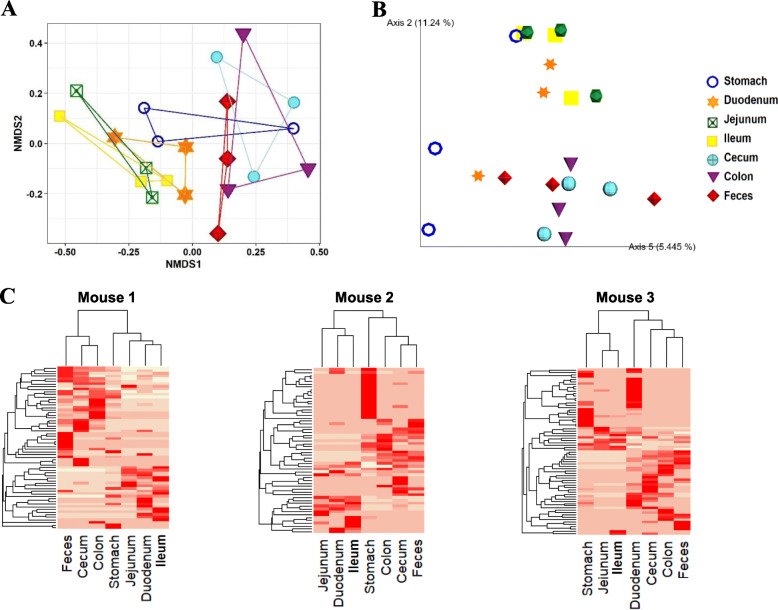
Comparison of microbial diversity at the different locations of the GI tract by β-diversity analysis. **a**. Non-metric multidimensional scaling (NMDS) plots showing the difference of microbiome in different locations of the GI tract at OTU level based on Bray-Curtis distances. **b**. Principal coordinate analysis (PCoA) based on the unweighted Unifrac metric of microbiome among all samples. The percentage of variation explained by PC2 and PC5 are indicated in the axis. **c**. Heatmap of the microbial composition and relative abundance of all samples based on the Bray–Curtis distance matrix calculated from relative OTU abundances at genus level

The discrepancy of the composition of gut microbiome along the GI tract became more evident with the ratio analysis between location and local species (Fig. 3, Figure S6-S8). To compare the diversities of the microbiomes at different locations, β analysis method was applied. The NMDS plots based on Bray-Curtis distances showed that the microbiome compositions were very diverse in location-specific manners in all of the tested three mice and that, more significantly, the fecal microbiome did not represent the microbiome of the GI tracts (R^2^ = 0.49, *P* = 0.003 ADONIS) (Fig. 3a, Figure S6A). We transformed the OTUs of each microbiome into principal components using an unweighted UniFrac metric for Principal coordinates analysis (PCoA). Eigenvalues of each microbiome in different locations of the GI tracts were very different from each other (Fig. 3b, Figure S6B, Figure S6C). PCoA confirmed again that the fecal microbiome communities in all of the tested three mice did not represent any part of the microbiome communities in the guts. Other ordination plot methods also clearly confirmed our result (Figure S7). The correlation analysis of OTU values with respect to the locations of the GI tract by drawing a heat map of the top-ranked OTUs defined at the bray curtis distance level revealed that feces had a distinguished microbial profile compared with any locations of the GI tracts (Fig. 3c, Figure S8). Unsupervised hierarchical clustering clearly partitioned the samples into two distinguished groups, and this pattern was observed repeatedly over a wide range of phylogenetic levels (Figure S8).

### Alpha-diversity analysis showed that the fecal microbiome did not represent the microbiome of the whole GI tract

**Fig. 4:**
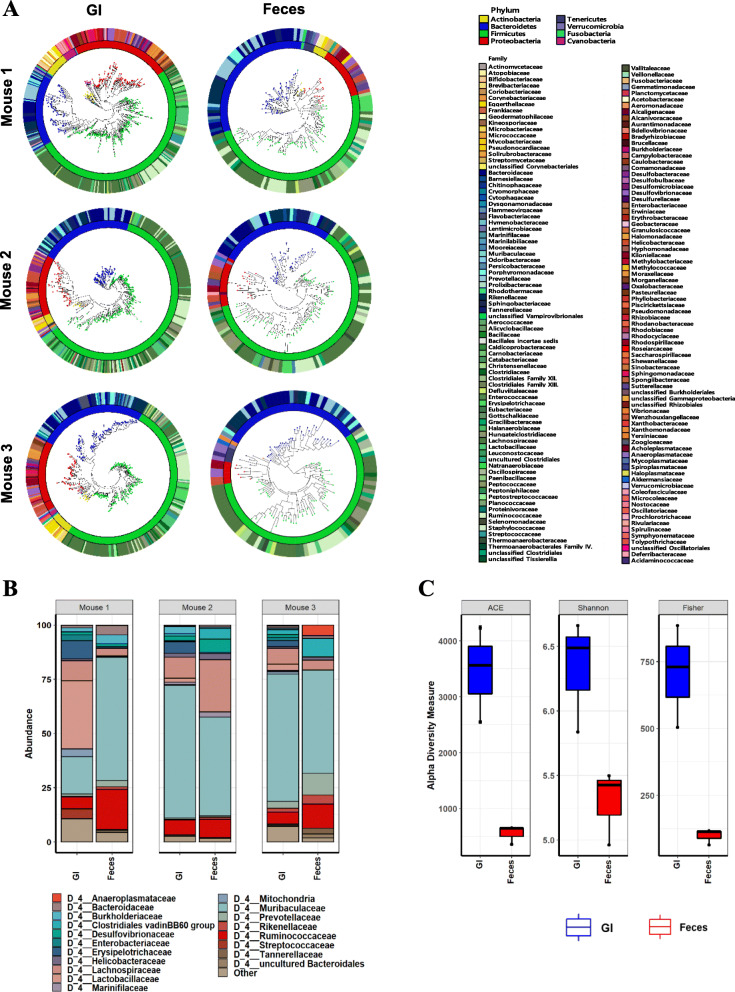
Comparative analysis on microbiome diversity in the GI tract and feces by α-diversity analyses. **a**. Maximum-likelihood phylogenetic tree comprising all of the taxa in the GI content and feces respectively. The rings of the circular dendrogram represent the family level and the corresponding phylum is depicted in the inner layer. **b**. Relative abundance of family level occupying in feces or the GI tract. **c**. Species richness and diversity measured by the indices of ACE richness, Shannon diversity and Fisher’s alpha

Location-specific analysis on microbiome clearly indicated that microbiome in the GI tract varied on its location under varied physical and chemical environments, and that fecal microbiome might not represent the actual microbiome in the GI tract (Figs. 1 and 2, Figure S2 ~ S8). To investigate that possibility, we directly compared fecal microbiome compositions with the microbiome composition of the whole GI tracts in each mouse. As expected, the gross microbiome analyses revealed that the microbiome composition of the GI tracts was clearly different from the the composition of the fecal microbiome (Fig. 4a, b, Figure S9A, S9B). The microbiome discrepancy between feces and the GI tract became more evident at lower taxonomic levels (Fig. 4b, Figure S9C). The most abundant microbial families in the GI tracts were Muribaculaceae, Lactobacillaceae, Lachnospiraceae Ruminococcaceae, and Erysipelotrichaceae in the decreasing order while Muribaculaceae, Ruminococcaceae, Lachnospiraceae, and Prevotellaceae were in fecal microbiomes (Fig. 4b). At the genus level*, Lactobacillus, Lactococcus, Dubosiella,* and *Turicibacter* were highly represented in the GI tract but not in feces (Figure S9D and Table S7).

After noting the microbiome discrepancy between feces and the GI tract by direct comparison, thorough taxonomic α-diversity analyses were performed. The taxonomic α-diversities measured by ACE richness, Shannon diversity, and Fisher’s alpha all indicated that the diversities of the microbiome of the GI tracts were much higher than the fecal microbiome (Fig. 4c). Also, the fecal microbiome did not represent the microbiome of the GI tracts in all of the three mice. Shannon diversity (*p* < 0 .05), ACE richness (*p* < 0.01), and Fisher’s alpha (p < 0.01) concluded that the microbiome of the GI tract was statistically different from the fecal microbiome and the fecal microbiome does not represent the microbiome of GI tract.

### Beta-diversity analysis confirmed that fecal microbiome did not represent the microbiome of the whole GI tract

**Fig. 5:**
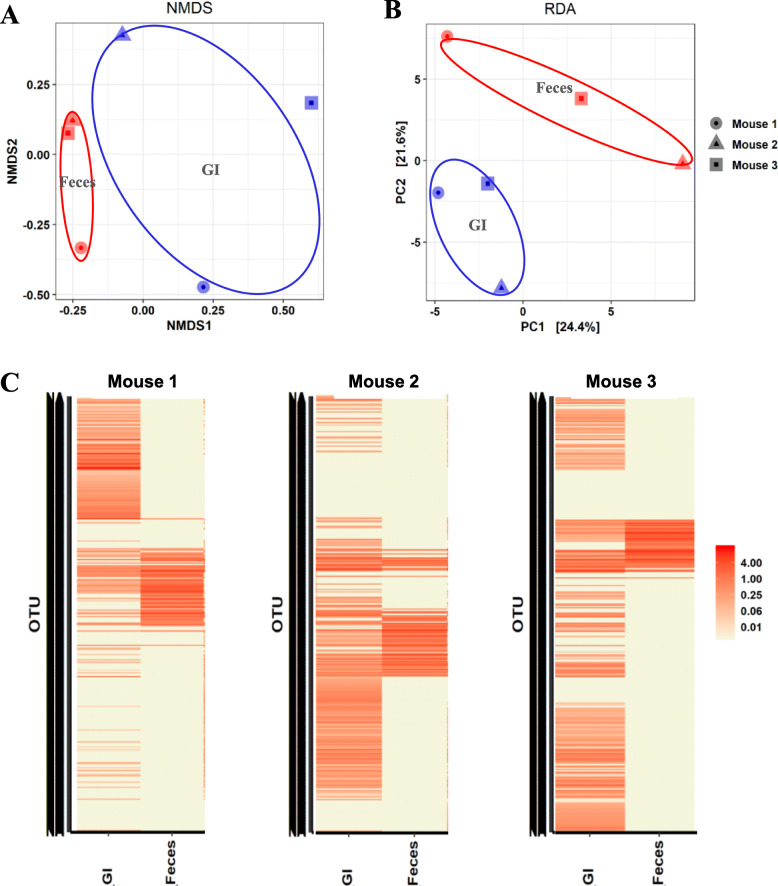
Comparative analysis of microbiome diversity in the GI tract and feces by β-diversity analysis. **a**. The NMDS plot showing the difference of microbiome between feces and the GI tract at OTU level based on Bray-Curtis distances. The 2D stress was 0.109. **b**. The RDA plot showing the difference of microbiome between feces and the GI tract at OTU level based on Bray-Curtis distances. **c**. Heatmap of the microbial composition and relative abundance of all samples based on the Bray–Curtis distance matrix calculated from relative OTU abundances at genus level

Comparative analysis on the microbiome of feces and the GI tracts by β diversity analyses (community structure: R^2^ = 0.1, p < 0 .05 ADONIS) further solidified that the fecal microbiome did not represent the microbiome in the GI tract of its host. Both NMDS and RDA plots showed that the fecal microbiome was distinctly different from the microbiome of the GI tracts in all of the tested mice (Fig. 5a, b). Interestingly, the microbial community of the fecal microbiome was closer to each other in individual mice than to that of the GI microbiome within the same mice. The distinct difference of microbiome compositions between feces and the GI tract within a mouse became more evident with a correlation analysis of total OTUs with respect to feces and the GI tract. The heat map of all OTUs defined at the Bray-Curtis distance level revealed that the fecal microbiome was completely different from that of the GI tracts as demonstrated in a distinguished pattern of microbial profile among feces and also among the GI tracts in all tested mice rather than between the microbiome compositions of feces and the GI tract within same mice (Fig. 5c).

## Discussion

The gut is the place where food is broken down and metabolized, nutrients are absorbed, water and minerals absorbed, waste metabolites are excreted, and pH and oxygen levels fluctuate [21]. These activities in gut are precisely regulated and thereby location-specific, which means that the environment condition inside gut is different location by location [22–26]. Accordingly, the chemical and physical compositions of feces differ from the GI contents in the large intestine [27]. In fact, this work showed that the pH and water content of feces even differed from those of the GI contents in the large intestine [28]. The pH differences noted here are well-matched with previous reports that the intraluminal pH at different locations ranged from 1.0 ~ 2.5 (stomach) to 6.6 ± 0.5 (large intestine) while the pH of feces was 7.5 ± 0.4 [18]. Considering these differences, it would not be surprising to note that the fecal microbiome is not a representative of the actual gut microbiome of its host because the growth and propagation of microbial organisms, including the intestinal microbes of mammals, depend on their surrounding environments.

Although the fact that the fecal microbiome differs from gut microbiome has not been recognized, there have been growing concerns regarding using feces as a proxy to study the gut microbiome. Yan et al. found a certain degree of discrepancy between the fecal microbiome and the gut microbiome in chicken [29]. It has been reported that stool sampling affects the heterogeneity and inconsistency of the fecal microbiome [28, 30, 31]. This works showed that even the microbiome in the GI content of the large intestine is different from the fecal microbiome (Figs. 1 and 2). Since the stool excretion from the large intestine can be influenced by various conditions, the heterogeneity and inconsistency of the fecal microbiome could be expected. Therefore, this work seems to well explain the previous works which accounted for the heterogeneity and inconsistency of fecal microbiome as stool inconsistency [28, 30, 31].

Although the validity of the fecal microbiome as a proxy of gut microbiome has been questioned previously, the question never been seriously investigated. Rather, fecal samples have been customarily used for investigation of gut microbiome after neglecting the fact that even stool sampling generates heterogeneity and inconsistency in the fecal microbiome. To compare the gut microbiome and fecal microbiome in the same condition, we used genetically homogenous sibling male mice grown in a co-housed condition to ensure that the experimental condition is identical for each mouse. Thorough statistical analyses showed that the microbiome in the GI tract is consistently changing reflecting environmental conditions at the location of the GI tract and thereby fecal microbiome is different from the whole gut microbiome of the GI tract.

While numerous recent research successfully showed that gut microbiome plays determinant roles in various phenotypes and diseases of its host [10, 32–34], those research are largely associative in nature and may fail to pinpoint the causative intestinal microbes for the phenotypes or diseases along with difficulties in consistency and reproduction by other researchers [35–37].

## Conclusions

This work suggests that the composition of gut microbiome differs in a location-specific manner and thereby fecal microbiome is just a part of the whole gut microbiome. Therefore, it would be reasonable to develop methodologies investigating the whole gut microbiome of its hosts such as detecting a blood signature of gut microbiome based on its adaptive immune-based signature, developing an endoscopic method for GI content sampling, etc.

## Methods

### Animals and sample collection

All animal care and use protocols were performed strictly in accordance with the ethical guidelines of the Ethics Committee of the Chonbuk National University Laboratory Animal Center (Permit Number: CBU 2012–0040) in accordance with the ‘Guide for the Care and Use of Laboratory Animals.

Six-week-old male C57BL/6 mice (Joongang Experimental Animal Co., Seoul, Korea) were purchased and acclimatized for 4 weeks. During the experimental period, the mice were housed in an animal room under controlled environmental conditions at a temperature of 22 ± 2 °C, relative humidity of 50 ± 5%, and a 12-h light/dark cycle, with a normal chow food and water readily available. The mice were transferred to freshly sterilized separate cages every morning to avoid coprophagy. When the mice reached ten weeks old, the feces were collected from sterile cages without bedding within two hours, and the mice were sacrificed by cervical dislocation. After sacrificing the mice, the whole GI tracts were segmented immediately into stomach, duodenum, jejunum, ileum, cecum, and colon according to the anatomical feature (Figure S1). The segments were subsequently opened along their cephalocaudal axis using a sterile scissor, and the GI contents in each segment were thoroughly harvested by collecting and followed by sampling with spatula. Each sample, except for pH measurement, was weighed and immediately frozen in liquid nitrogen and were stored at -80C until DNA extraction.

### Determination of the water contents of the GI contents and feces

The water contents of each GI contents and feces were determined by subtracting dry weights from the wet weights. The wet weights of the all samples were measured before lyophilization and the dry masses were measured after lyophilization.

### pH determination of the GI contents

Approximately 0.1 g of each GI content was transferred into Eppendorf tube containing 0.9 ml ddH_2_O. After thorough mixing followed by standing 1 h at room temperature, pH of each sample was measured, using a pre-calibrated Orion Star™ A210 series benchtop pH meter (Fisher Scientific). pH was measured three times and averaged.

### Microbiome DNA preparation

Total genomic DNA from each sample was extracted using the phenol-chloroform isoamyl alcohol extraction protocol, as described previously [38]. Briefly, lysis buffer (200 mM NaCl, 200 mM Tris-HCl (pH 8.0), 20 mM EDTA) suspended samples were processed by bead beating, and the genomic DNA recovered from aqueous phase by phenol:chloroform:isoamylalcohol. DNA precipitated with the addition of 3 M sodium acetate followed by isopropanol. After rinsing with 70% ethanol and drying, the DNA pellet was dissolved in TE buffer (10 mM Tris-HCl pH 8.0, 1 mM EDTA). DNA was quantified using a BioSpec-nano spectrophotometer (Shimadzu Biotech).

### Bacterial 16 s rDNA genes sequencing

The sequencing samples are prepared according to the Illumina 16S rDNA Metagenomic Sequencing Library protocols. The 16S rDNA genes were amplified using 16S rDNA V3-V4 primers (16S rDNA Amplicon PCR Forward Primer: 5′ TCGTCGGCAGCGTCAGATGTGTATAAGAGACA GCCTACGGGNGGCWGCAG; 16S rDNA Amplicon PCR Reverse Primer: 5′ GTCTCGTGGGC TCGGAGATGTGTATAAGAGA CAGGACTACHVGGGTATCTAATCC). Input gDNA was amplified with 16S rDNA V3-V4 primers, and a subsequent limited cycle amplification step was performed to add multiplexing indices and Illumina sequencing adapters [39]. The final products were normalized and pooled using the PicoGreen, and the size of libraries were verified using the TapeStation DNA screentape D1000 (Agilent). And sequencing (2 × 300) was done using the MiSeq™ platform (Illumina) according to the standard protocol.

### Sequencing data analysis

To improve genome assembly, the paired-end reads from NGS (Next Generation Sequencing) were merged using FLASH (Fast Length Adjustment of Short reads) [40]. The amplicon error was modelled from merged fastq using DaDa2 and filtered out noise sequence, corrected errors in marginal sequences, removed chimeric sequences, removed singleton, and then dereplicated those sequences [41]. In this study, we used denoise-single function that set as default parameter. The Q2-Feature classifier is a Naive Bayes classifier trained based on SILVA reference (region V3-V4) database (https://www.arb-silva.de/) to classify the dataset used in the experiment [42]. The q2-diversity used with “sampling-depth” option in the diversity calculation and statistical tests [43]. After checking the data in the “table.qzv” file, feature count was filtered by setting the threshold according to the experiment in QIIME 2 [43].

### Data preprocessing

The metagenome sequence data of each sample was analyzed by using the phyloseq package (1.28.0) in R version 3.6.1 [44]. Taxanomy classification table, OTU, and metadata, were imported as phyloseq object. The OTUs that are not presents in at least one sample were removed, as considered as sequencing errors. The data was normalized by the cumulative-sum-scaling (CSS) using the metagenomeSeq (1.16.0.) package from Bioconductor software [45]. Further analysis, and visualization was done by using the phyloseq package.

### Evaluation of alpha diversity and relative abundance of microbiome

The CSS normalized values were used to calculate the alpha diversity (ACE richness, Fisher’s alpha, Inverse Simpson, Simpson and Shannon diversity, and Evenness) metrics in phyloseq package without filtering [46]. To detect differences in richness and alpha diversity between groups, we used Kruskal-Wallis rank sum test., and filtered data was converted into relative abundance. Further, unclassified phyla were removed from total samples. Any taxa with a total of less than 0.5% were collapsed into “other” and each taxanomy level was calculated before plotting.

### Evaluation of Beta diversity of microbiome

Beta diversity metrics were computed and visualized using log transformed, normalized OTU data in phyloseq package including Bray-Curtis dissimilarity [47]. Permutational multivariate analysis of variance (PERMANOVA) was applied to identify statistical significance of beta diversity between groups by using the vegan package in R. ADONIS was used with 999 permutations in the vegan package in R to quantify the effect size of variables explaining Bray-Curtis distance [48]. Unweighted PCoA was calculated and visualized by QIIME2, however; NMDS, RDA, MDS, CCA, and DCA were plotted in the phyloseq package in R.

### Construction of heatmap and phylogenetic tree

The core abundant OTU values at genus level were used to generate a heatmap and cluster analysis by using the Heatplus (2.30.0.) package from Bioconductor. The OTUs obtained by *unsupervised* prevalence filtering after setting the 5% of total samples as the threshold were used to construct the most abundant taxonomies as a heatmap. The cluster analysis on the most abundant taxonomies was done by using Bray-Curtis distance metrix and average linkage hierarchical clustering, respectively [49].

Phylogenetic trees were constructed to visualize the sample richness, and all row sequences were used without filter to show direct relation to taxonomy. Taxonomizr (0.5.3) package in R was applied to reclassify the unclassified taxonomies based on the NCBI accession number [50]. Alignment for 16 s rDNA sequences was done by ClustalW [51] program with default parameter. Consequently, construction of the Maximum-likelihood phylogenetic trees were done in MEGAX [52] with 500 bootstraps replicates, and visualized by iTOL [53].

### Statistical analysis

All statistical analyses are reported as the mean ± SEM, and the differences in relative abundance of bacterial populations among feces to GI parts were analysed using the Mann-Whitney sum rank tests in R software. Significance was declared at *P* < 0.05. All graphs were prepared with R software.

## Supplementary Information

**Additional file 1: Figure S1**. The photo pictures of the whole GI tracts used in this experiment. (1) Stomach, (2) Duodenum, (3) Jejunum, (4) Ileum, (5) Cecum, (6) Colon. **Figure S2**. Maximum-likelihood phylogenetic tree comprising the taxa in each location of the GI tract of mouse number 1. The rings of the circular dendrogram represent the family level and the corresponding phylum is depicted in the inner layer and brunch node. (a) Feces, (b) Stomach, (c) Duodenum, (d) Jejunum, (e) Ileum, (f) Cecum, (g) Colon. **Figure S3**. Maximum-likelihood phylogenetic tree comprising the taxa in each location of the GI tract of mouse number 2. The rings of the circular dendrogram represent the family level and the corresponding phylum is depicted in the inner layer and brunch node. (a) Feces, (b) Stomach, (c) Duodenum, (d) Jejunum, (e) Ileum, (f) Cecum, (g) Colon. **Figure S4**. Maximum-likelihood phylogenetic tree comprising the taxa in each location of the GI tract of mouse number 3. The rings of the circular dendrogram represent the family level and the corresponding phylum is depicted in the inner layer and brunch node. (a) Feces, (b) Stomach, (c) Duodenum, (d) Jejunum, (e) Ileum, (f) Cecum, (g) Colon. **Figure S5**. Relative abundance of taxonomic groups of microorganisms occupying in the different GI sections and feces in each mouse. (a) Class, (b) Order, (c) Family, and (d) Genus levels. **Figure S6**. Comparison of microbial diversity at the different locations of the GI tract in the same mouse by β-diversity analysis. A. Non-metric multidimensional scaling (NMDS). B. 3D Principal coordinate analysis (PCoA). C. 2D Principal coordinate analysis (PCoA). The percentage of variation explained by indicated axis. **Figure S7**. Ordination plots based on the Bray-Curtis distances in the microbial communities of the GI tracts. 2D stress values were 0.03, 0.29, 0.086 and 0.14 for mouse 1, mouse 2, mouse 3 and all mice respectively. A. Redundancy analysis (RDA), B. ta (DCA), C. Multidimensional scaling (MDS) and D. Correspondence analysis (CA). **Figure S8**. Correlation of all OTU values at genus level. Heatmap of the microbial composition and relative abundance of all samples based on the Bray–Curtis distance matrix calculated from relative OTU abundances at genus level. **Figure S9**. Relative abundance of taxonomic groups of microorganisms occupying in the GI tract and feces in each mouse. (a) Phylum, (b) Class, (c) Order, (d) Genus levels.

**Additional file 2: Table S1**. The water contents in each location of the GI contents. **Table S2**. The pH measurements in each location of the GI contents. **Table S3**. Abundance of phyla level bacterial taxa in the GI sections and Feces (mean ± SEM, % of assigned 16S rRDA gene sequences) Taxa above 1% abundance are written in a bold format. **Table S4**. Abundance of class level bacterial taxa in the GI sections and Feces (mean ± SEM, % of assigned 16S rRDA gene sequences) Taxa above 1% abundance are written in a bold format. **Table S5**. Abundance of order level bacterial taxa in the GI sections and Feces (mean ± SEM, % of assigned 16S rRDA gene sequences) Taxa above 1% abundance are written in a bold format. **Table S6**. Abundance of family level bacterial taxa in the GI sections and Feces (mean ± SEM, % of assigned 16S rRDA gene sequences) Taxa above 1% abundance are written in a bold format. **Table S7**. Abundance of genus level bacterial taxa in the GI sections and Feces (mean ± SEM, % of assigned 16S rRDA gene sequences) Taxa above 1% abundance are written in a bold format. **Table S8**. Overview of metagenomics sequencing results for each sample. Numerical numbers 1 ~ 3 indicate mouse numbers used in this experiment.

## Data Availability

The datasets used and/or analysed during the current study available from the corresponding author on reasonable request.

## Abbreviations

CCA: canonical correspondence analysis
CSS: cumulative-sum-scaling
DCA: detrended correspondence analysis
FLASH: fast length adjustment of short reads
gDNA: genomic deoxyribonucleic Acid
GI: gastrointestinal
IBD: inflammatory bowel disease
MDS: multidimensional scaling
NGS: next generation sequencing
NMDS: non-metric multidimensional scaling
OTU: operational taxonomic unit
PCoA: principal coordinates analysis
PCR: Polymerase chain reaction
RDA: redundancy analysis
rRNA: ribosomal ribonucleic acid
16S rDNA: 16S ribosomal deoxyribonucleic acid
