# Supplementary material for: The regional diversity of gut microbiome along the GI tract of male C57BL/6 mice
Source: BMC Microbiol. 2021 Feb 12;21:44. doi: 10.1186/s12866-021-02099-0 (PMC7881553; doi:10.1186/s12866-021-02099-0)

The regional diversity of gut microbiome along the GI tract of male C57BL/6 mice

**Additional file 1**

**Figure S1**.


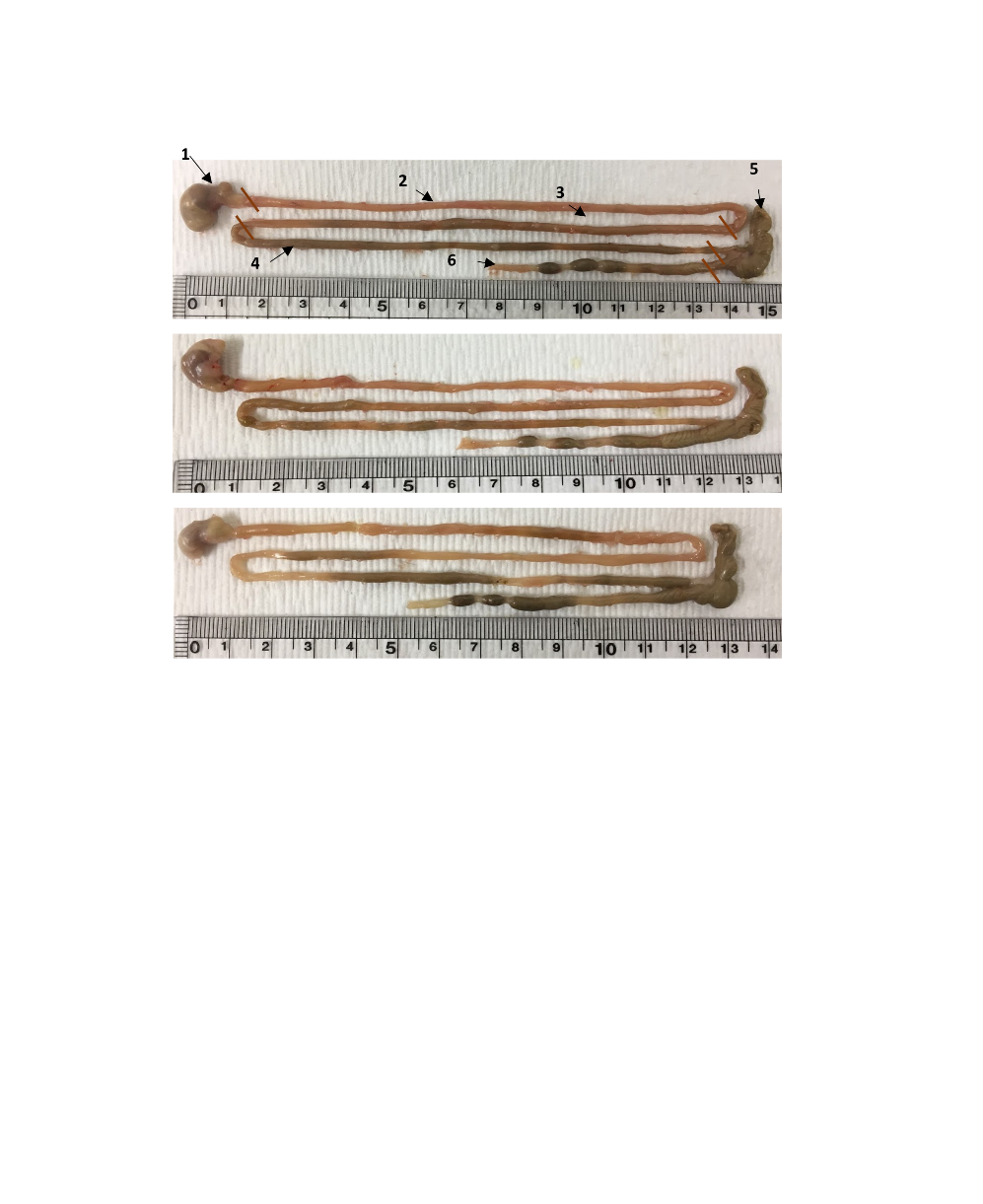


**Figure S2.**

**
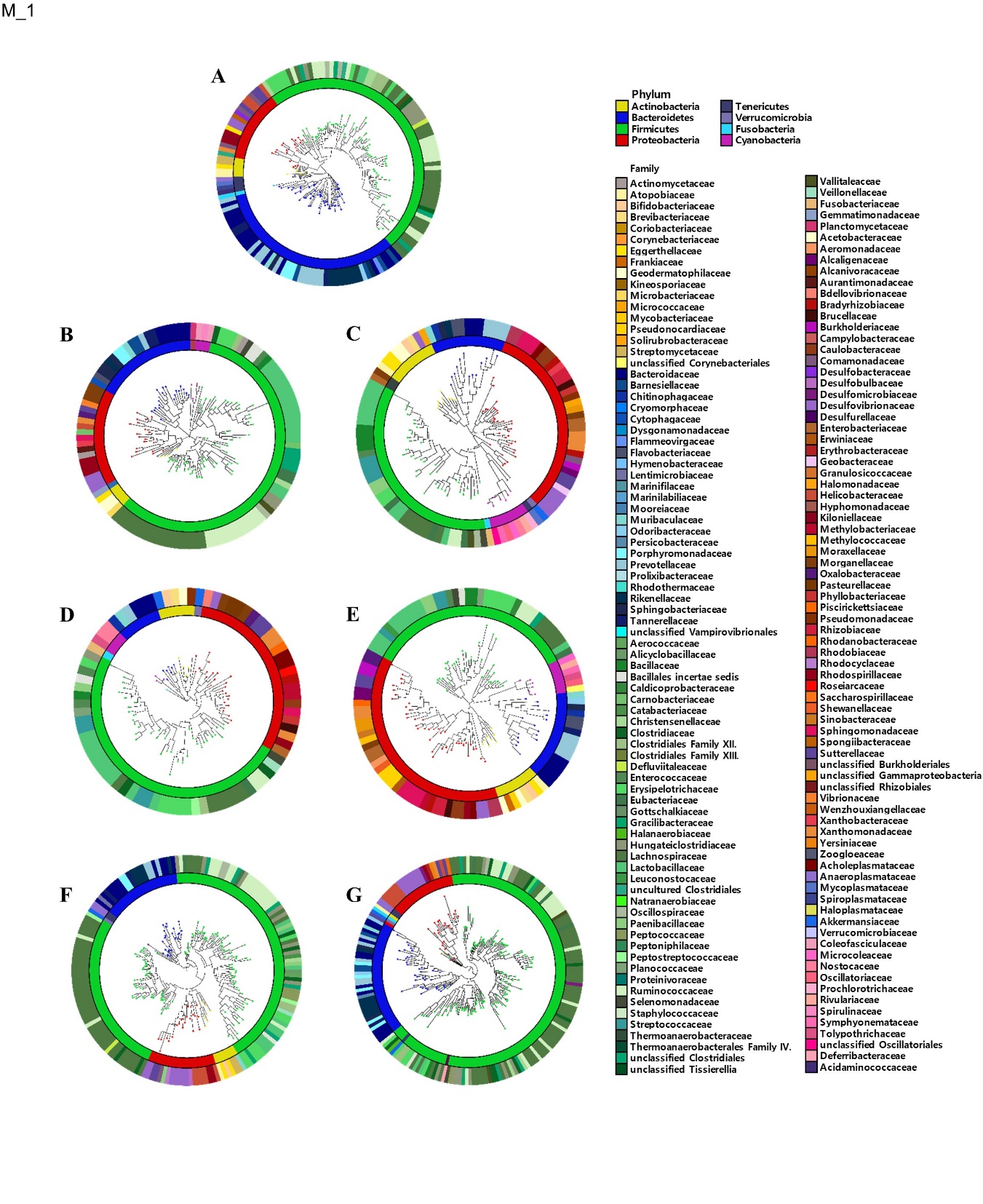
**

**Figure S3.**

**
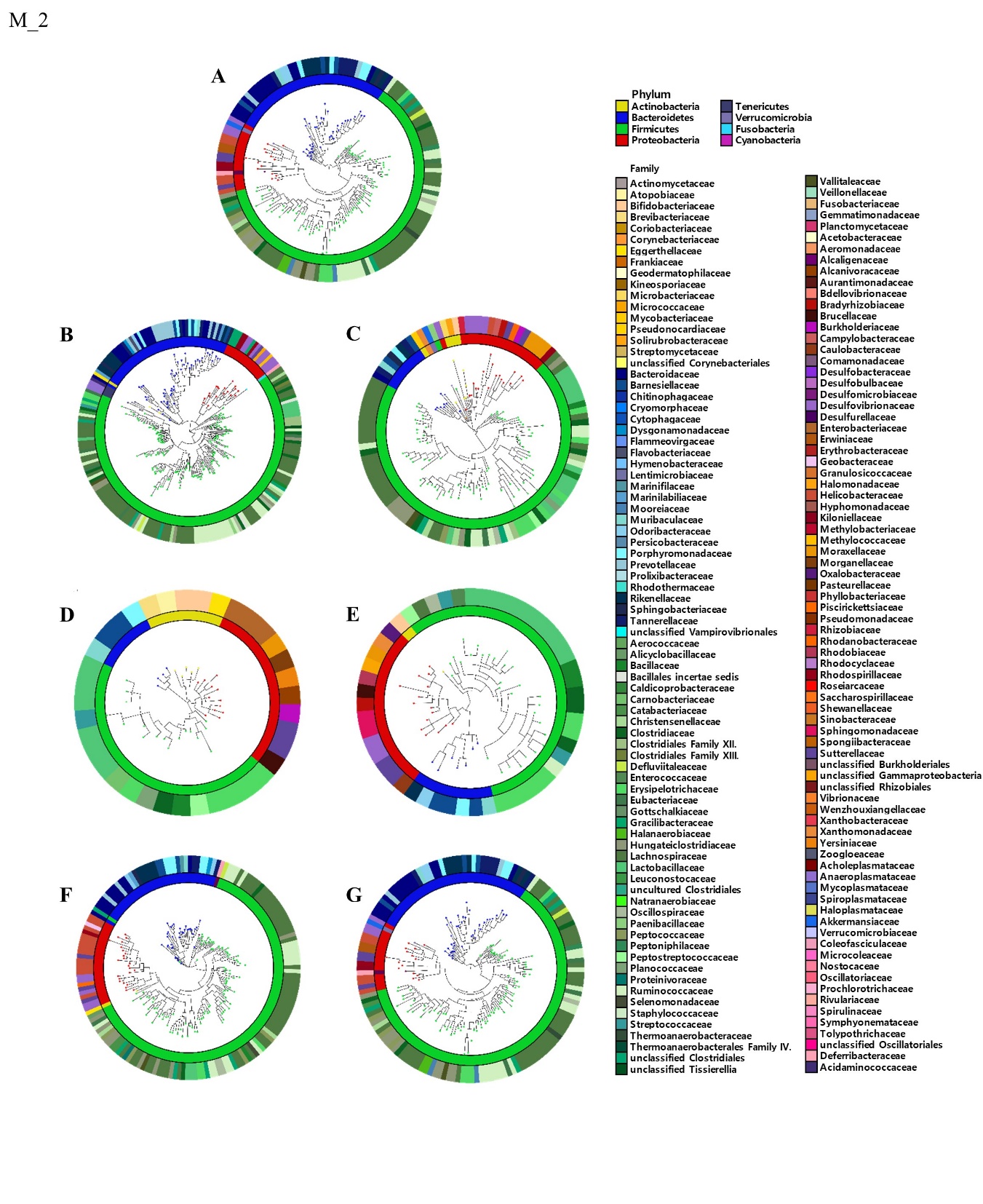
.**

**Figure S4.**


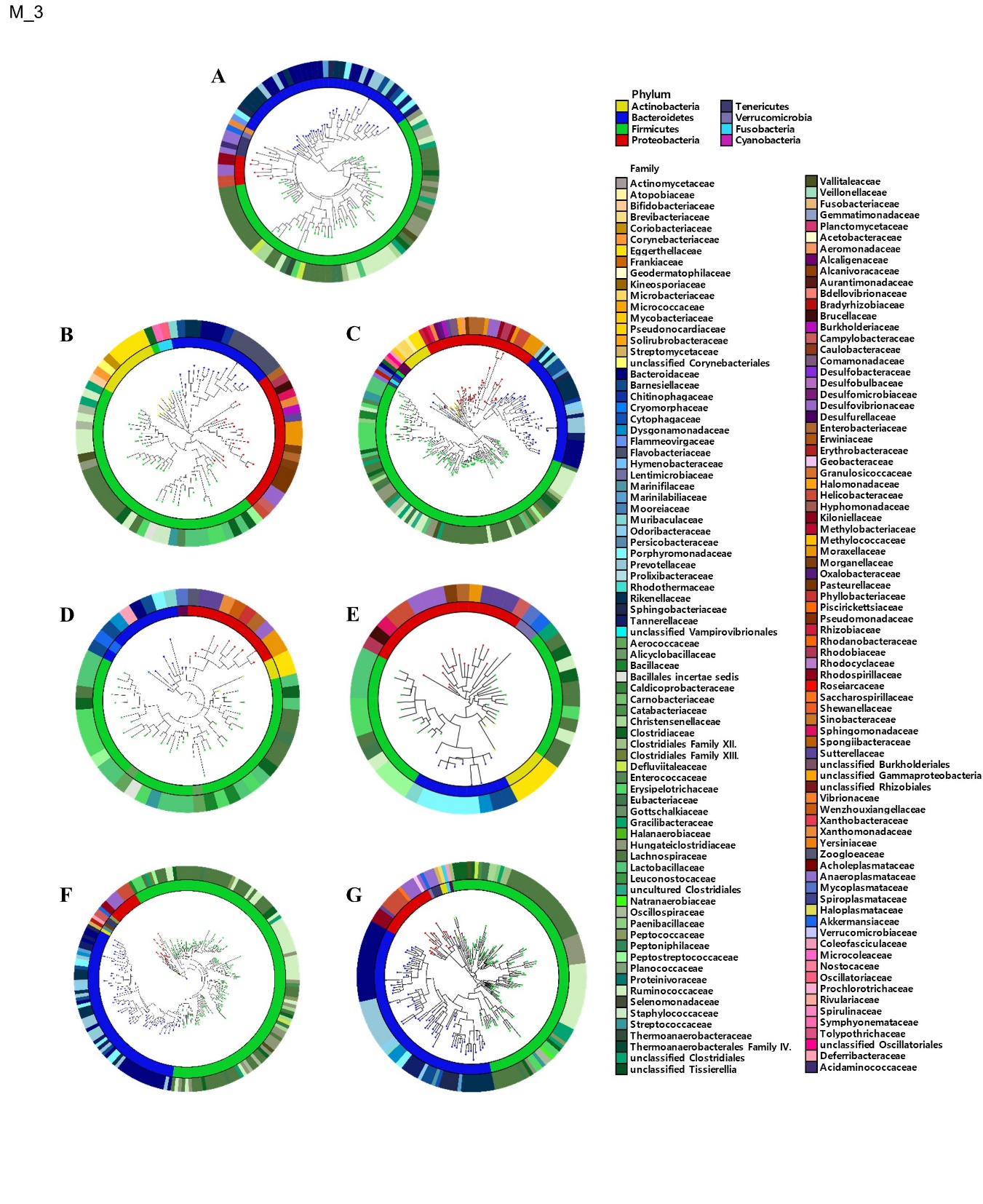


**Figure S5.**


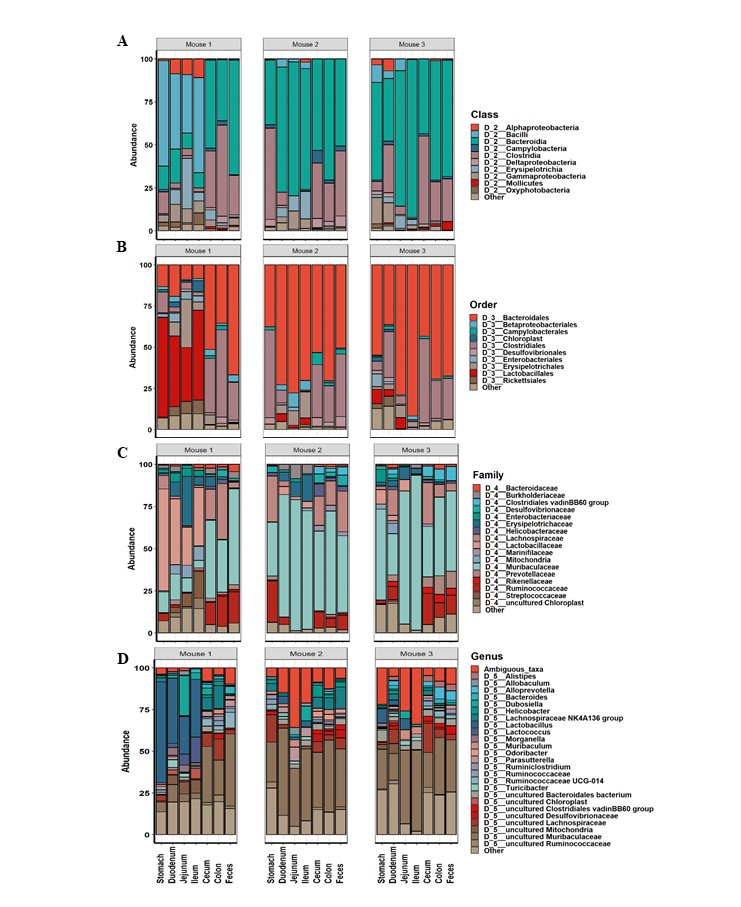


**Figure S6.**

**
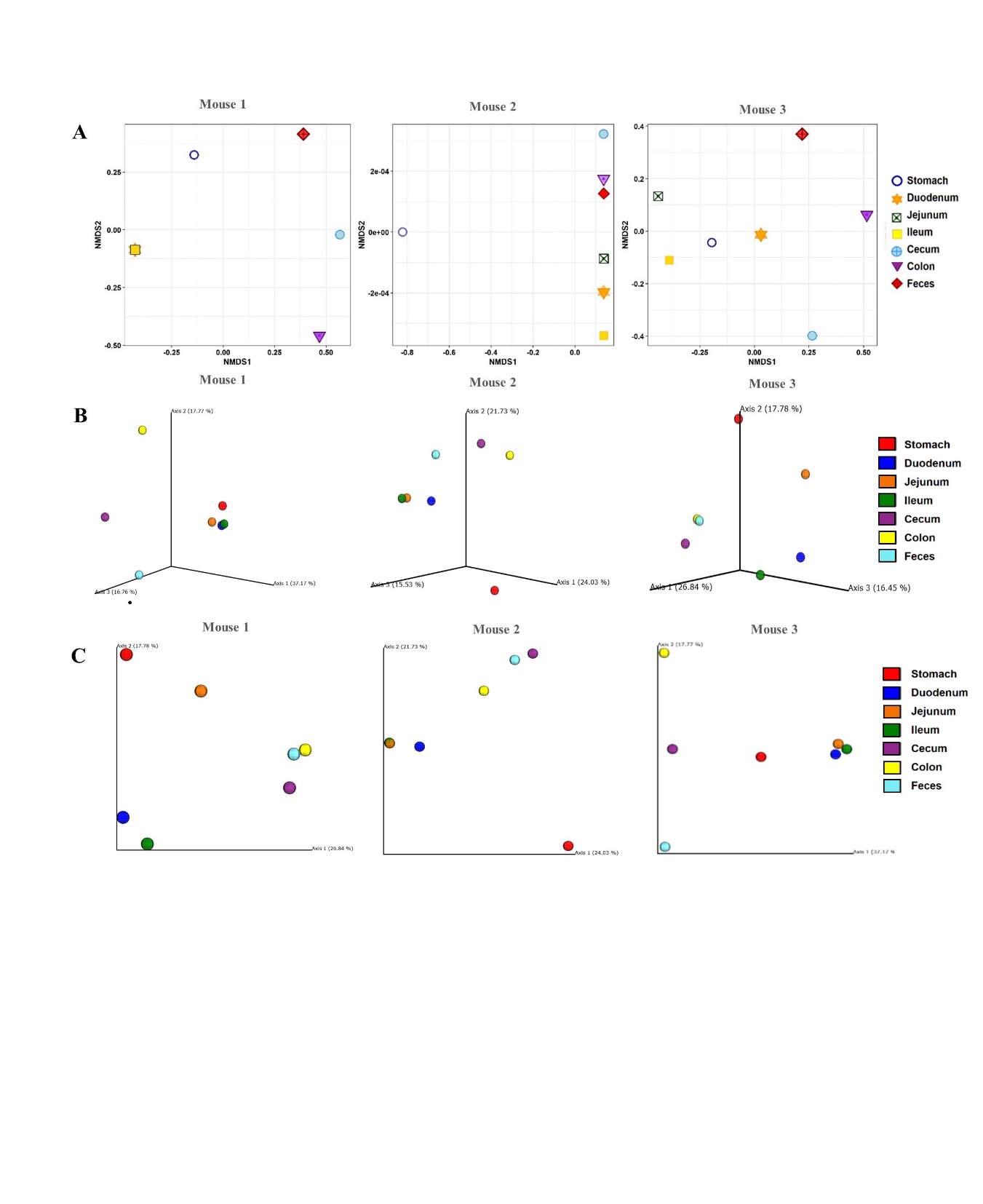
**

**Figure S7.**

**
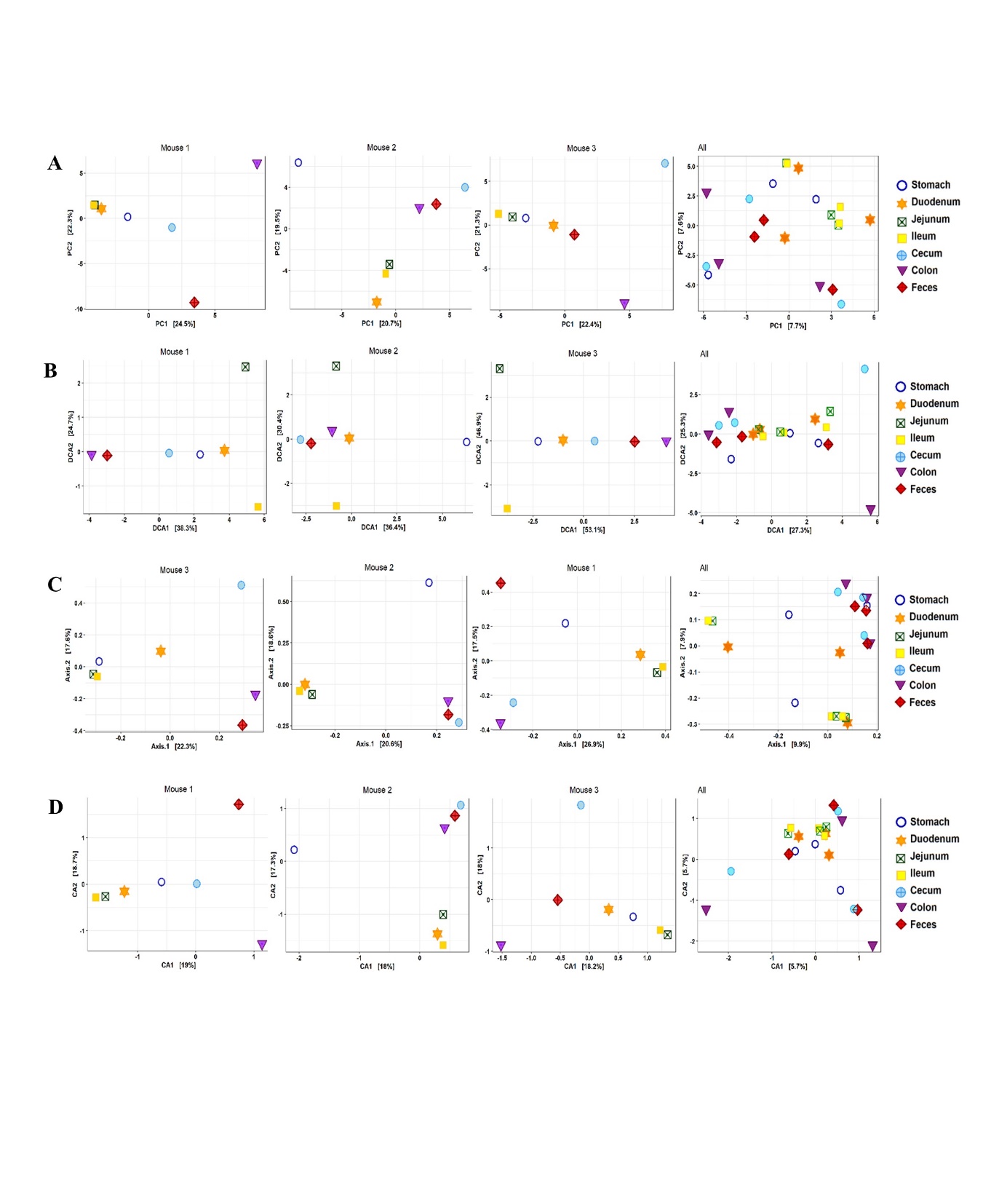
**

**Figure S8**.


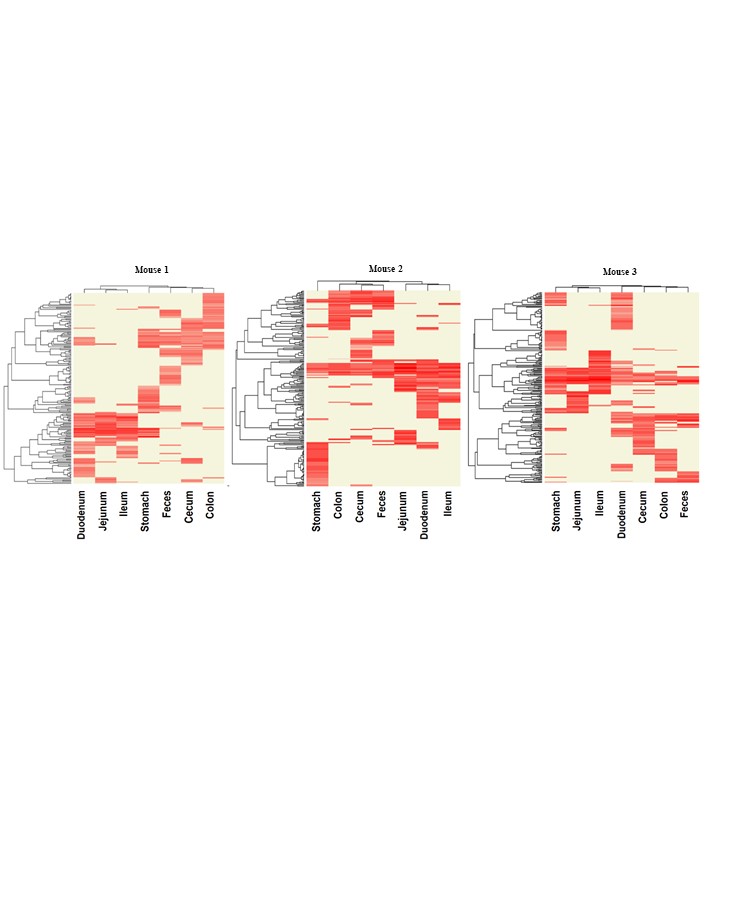


**Figure S9**.


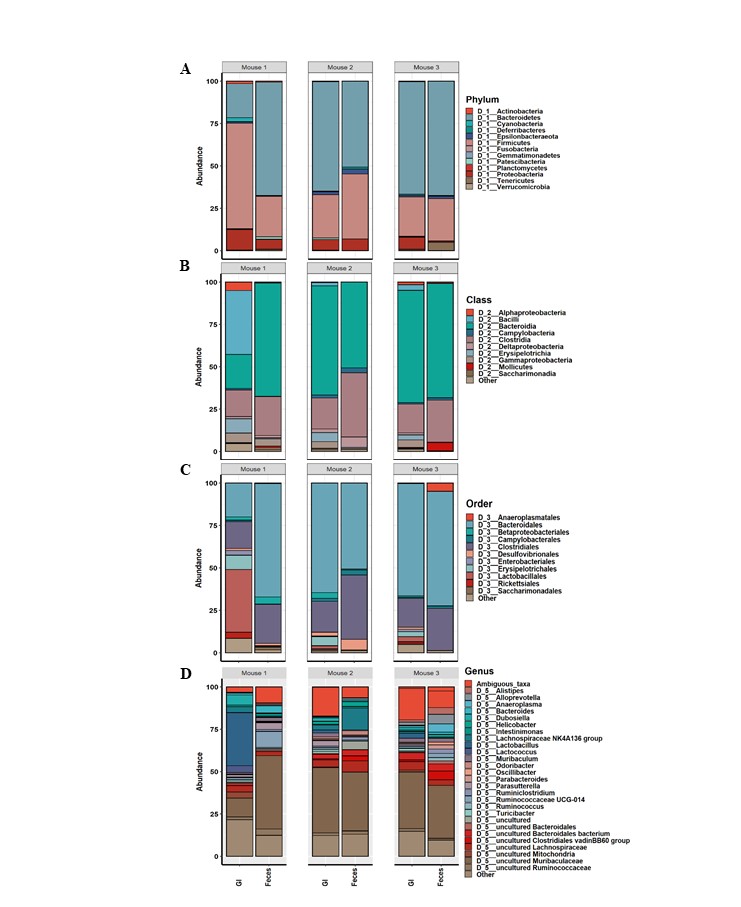

Supplement: Supplementary file 1 — Additional file 1: Figure S1. The photo pictures of the whole GI tracts used in this experiment. (1) Stomach, (2) Duodenum, (3) Jejunum, (4) Ileum, (5) Cecum, (6) Colon. Figure S2. Maximum-likelihood phylogenetic tree comprising the taxa in each location of the GI tract of mouse number 1. The rings of the circular dendrogram represent the family level and the corresponding phylum is depicted in the inner layer and brunch node. (a) Feces, (b) Stomach, (c) Duodenum, (d) Jejunum, (e) Ileum, (f) Cecum, (g) Colon. Figure S3. Maximum-likelihood phylogenetic tree comprising the taxa in each location of the GI tract of mouse number 2. The rings of the circular dendrogram represent the family level and the corresponding phylum is depicted in the inner layer and brunch node. (a) Feces, (b) Stomach, (c) Duodenum, (d) Jejunum, (e) Ileum, (f) Cecum, (g) Colon. Figure S4. Maximum-likelihood phylogenetic tree comprising the taxa in each location of the GI tract of mouse number 3. The rings of the circular dendrogram represent the family level and the corresponding phylum is depicted in the inner layer and brunch node. (a) Feces, (b) Stomach, (c) Duodenum, (d) Jejunum, (e) Ileum, (f) Cecum, (g) Colon. Figure S5. Relative abundance of taxonomic groups of microorganisms occupying in the different GI sections and feces in each mouse. (a) Class, (b) Order, (c) Family, and (d) Genus levels. Figure S6. Comparison of microbial diversity at the different locations of the GI tract in the same mouse by β-diversity analysis. A. Non-metric multidimensional scaling (NMDS). B. 3D Principal coordinate analysis (PCoA). C. 2D Principal coordinate analysis (PCoA). The percentage of variation explained by indicated axis. Figure S7. Ordination plots based on the Bray-Curtis distances in the microbial communities of the GI tracts. 2D stress values were 0.03, 0.29, 0.086 and 0.14 for mouse 1, mouse 2, mouse 3 and all mice respectively. A. Redundancy analysis (RDA), B. ta (DCA), C. Multidimensional scali [file 12866_2021_2099_MOESM1_ESM.docx]
