# Supplementary material for: The regional diversity of gut microbiome along the GI tract of male C57BL/6 mice
Source: BMC Microbiol. 2021 Feb 12;21:44. doi: 10.1186/s12866-021-02099-0 (PMC7881553; doi:10.1186/s12866-021-02099-0)
Supplement: Supplementary file 2 — Additional file 2: Table S1. The water contents in each location of the GI contents. Table S2. The pH measurements in each location of the GI contents. Table S3. Abundance of phyla level bacterial taxa in the GI sections and Feces (mean ± SEM, % of assigned 16S rRDA gene sequences) Taxa above 1% abundance are written in a bold format. Table S4. Abundance of class level bacterial taxa in the GI sections and Feces (mean ± SEM, % of assigned 16S rRDA gene sequences) Taxa above 1% abundance are written in a bold format. Table S5. Abundance of order level bacterial taxa in the GI sections and Feces (mean ± SEM, % of assigned 16S rRDA gene sequences) Taxa above 1% abundance are written in a bold format. Table S6. Abundance of family level bacterial taxa in the GI sections and Feces (mean ± SEM, % of assigned 16S rRDA gene sequences) Taxa above 1% abundance are written in a bold format. Table S7. Abundance of genus level bacterial taxa in the GI sections and Feces (mean ± SEM, % of assigned 16S rRDA gene sequences) Taxa above 1% abundance are written in a bold format. Table S8. Overview of metagenomics sequencing results for each sample. Numerical numbers 1 ~ 3 indicate mouse numbers used in this experiment. [file 12866_2021_2099_MOESM2_ESM.docx]

**The regional diversity of gut microbiome along the GI tract of male C57BL/6 mice**

**Additional file 2**

**Table S1**. The water contents in each location of the GI contents

| **Locations** | **Water contents (%)** |
| --- | --- |
| Stomach | 66.43 |
| Duodenum | 75.56 |
| Jejunum | 73.58 |
| Ileum | 81.25 |
| Cecum | 76.16 |
| Colon | 78.13 |
| Feces | 48.48 |

**Table S2.** The pH measurements in each location of the GI contents

| **Locations** | **pH** |
| --- | --- |
| Stomach | 3.99±0.25 |
| Duodenum | 6.20±0.29 |
| Jejunum | 6.79±0.26 |
| Ileum | 7.25±0.21 |
| Cecum | 6.93±0.22 |
| Colon | 6.96±0.22 |
| Feces | 7.02±1.25 |

**Table S3.** Abundance of phyla level bacterial taxa in the GI sections and Feces (mean±SEM, % of assigned 16S rRNA gene sequences) Taxa above 1% abundance are written in a bold format.

| **Phylum** | **Stomach** | **Duodenum** | **Jejunum** | **Ileum** | **Cecum** | **Colon** | **Feces** | **GI** |
| --- | --- | --- | --- | --- | --- | --- | --- | --- |
| Bacteroidetes | 35.95±10.20 | 43.02±12.76 | 55.19±18.86 | 56.99±20.28 | 49.28±2.46 | 58.43±9.38 | 61.67±4.49 | 49.81±3.31 |
| Firmicutes | 48.54±13.53 | 36.44±8.91 | 32.58±14.52 | 29.41±14.41 | 41.30±4.44 | 33.84±8.63 | 29.09±3.82 | 37.02±2.58 |
| Proteobacteria | 10.28±4.30 | 15.09±4.20 | 10.39±4.04 | 9.61±3.63 | 4.33±0.98 | 3.83±0.76 | 4.52±1.54 | 8.92±1.58 |
| Epsilonbacteraeota | 1.42±0.26 | 0.68±0.00 | 0 | 0.01±0.00 | 3.46±1.55 | 1.75±0.36 | 1.50±0.62 | 1.22±0.48 |
| Cyanobacteria | 1.74±0.61 | 1.50±0.48 | 0.34±0.00 | 2.25±0.00 | 0.21±0.01 | 0.39±0.16 | 0.19±0.01 | 1.07±0.32 |
| Actinobacteria | 0.69±0.30 | 0.86±0.35 | 1.29±0.71 | 1.23±0.55 | 0.13±0.10 | 0.02±0.00 | 0.21±0.18 | 0.70±0.20 |
| Patescibacteria | 0.71±0.32 | 1.60±0.87 | 0.07±0.03 | 0.04±0.00 | 0.02±0.00 | 0.59±0.45 | 0.51±0.00 | 0.51±0.23 |
| Deferribacteres | 0.09±0.00 | 0.13±0.00 | 0 | 0 | 0.26±0.14 | 0.34±0.25 | 0.40±0.00 | 0.14±0.04 |
| Tenericutes | 0.20±0.08 | 0.32±0.20 | 0.01±0.00 | 0.21±0.13 | 0.94±0.26 | 0.77±0.09 | 1.90±1.24 | 0.41±0.14 |
| Planctomycetes | 0.30±0.00 | 0 | 0 | 0 | 0 | 0 | 0 | 0.05±0.00 |
| Verrucomicrobia | 0.004±0.00 | 0.30±0.12 | 0.10±0.01 | 0.10±0.00 | 0.07±0.02 | 0.04±0.02 | 0.01±0.00 | 0.10±0.04 |
| Fusobacteria | 0 | 0.06±0.00 | 0.04±0.00 | 0.14±0.00 | 0 | 0 | 0 | 0.04±0.02 |
| Gemmatimonadetes | 0.08±0.00 | 0 | 0 | 0 | 0 | 0 | 0 | 0.01±0.00 |

**Table S4.** Abundance of class level bacterial taxa in the GI sections and Feces (mean±SEM, % of assigned 16S rRNA gene sequences) Taxa above 1% abundance are written in a bold format.

| **Class** | **Stomach** | **Duodenum** | **Jejunum** | **Ileum** | **Cecum** | **Colon** | **Feces** | **GI** |
| --- | --- | --- | --- | --- | --- | --- | --- | --- |
| Bacteroidia | 35.95±10.20 | 43.02±12.76 | 55.19±18.86 | 56.99±20.28 | 49.28±2.46 | 58.43±9.38 | 61.67±4.49 | 49.81±3.31 |
| Bacilli | 24.01±15.38 | 17.64±10.67 | 14.19±8.17 | 19.79±4.54 | 0.17±0.09 | 0.12±0.08 | 0.11±0.07 | 12.65±3.80 |
| Alphaproteobacteria | 1.57±0.79 | 5.20±2.15 | 3.07±2.48 | 4.28±2.71 | 0.13±0.05 | 0.45±0.27 | 0.37±0.12 | 2.45±0.77 |
| Deltaproteobacteria | 2.24±0.77 | 1.51±0.40 | 0.71±0.26 | 0.34±0.08 | 2.77±1.05 | 2.51±0.63 | 2.51±1.52 | 1.68±0.37 |
| Campylobacteria | 1.42±0.26 | 0.68±0.00 | 0 | 0.01±0.00 | 3.46±1.55 | 1.75±0.36 | 1.50±0.62 | 1.22±0.48 |
| Erysipelotrichia | 0.64±0.45 | 5.77±1.20 | 15.18±5.79 | 8.07±3.24 | 2.30±.46 | 1.00±0.26 | 0.41±0.12 | 5.49±2.07 |
| Clostridia | 23.81±12.10 | 12.89±6.24 | 3.04±1.20 | 1.27±0.41 | 38.74±.04 | 32.70±8.29 | 28.57±3.82 | 18.74±5.79 |
| Gammaproteobacteria | 6.46±3.71 | 8.37±2.01 | 6.62±2.37 | 4.99±1.11 | 1.35±1.05 | 0.87±0.36 | 1.63±1.02 | 4.77±1.13 |
| Oxyphotobacteria | 1.56±0.12 | 1.45±0.52 | 0.34±0.00 | 2.25±0.00 | 0 | 0 | 0 | 0.93±0.28 |
| Actinobacteria | 0.46±0.03 | 0.45±0.20 | 0.85±0.26 | 0.84±0.00 | 0.01±0.00 | 0 | 0.002±0.00 | 0.43±0.13 |
| Saccharimonadia | 0.71±0.32 | 1.60±0.87 | 0.02±0.00 | 0.04±0.00 | 0.02±0.00 | 0.59±0.45 | 0.51±0.00 | 0.50±0.23 |
| Deferribacteres | 0.09±0.00 | 0.13±0.00 | 0 | 0 | 0.26±0.14 | 0.34±0.25 | 0.40±0.00 | 0.14±0.04 |
| Mollicutes | 0.20±0.08 | 0.32±0.20 | 0.01±0.00 | 0.21±0.13 | 0.94±0.26 | 0.77±0.09 | 1.90±1.24 | 0.41±0.14 |
| Coriobacteriia | 0.23±0.16 | 0.41±0.16 | 0.44±0.33 | 0.38±0.31 | 0.13±0.10 | 0.02±0.00 | 0.21±0.00 | 0.27±0.06 |
| Planctomycetacia | 0.30±0.00 | 0 | 0 | 0 | 0 | 0 | 0 | 0.05±0.00 |
| Verrucomicrobiae | 0.004±0.00 | 0.30±0.12 | 0.10±0.01 | 0.10±0.00 | 0.07±0.02 | 0.04±0.02 | 0.01±0.00 | 0.10±0.04 |
| Negativicutes | 0.05±0.00 | 0.11±0.00 | 0.12±0.00 | 0.25±0.00 | 0 | 0.004±0.00 | 0 | 0.09±0.03 |
| Fusobacteriia | 0 | 0.06±0.00 | 0.04±0.00 | 0.14±0.00 | 0 | 0 | 0 | 0.04±0.02 |
| Melainabacteria | 0.18±0.01 | 0.06±0.00 | 0 | 0 | 0.21±0.01 | 0.39±0.16 | 0.19±0.01 | 0.14±0.05 |
| Gemmatimonadetes | 0.08±0.00 | 0 | 0 | 0 | 0 | 0 | 0 | 0.01±0.00 |
| Gracilibacteria | 0 | 0 | 0.05±0.00 | 0 | 0 | 0 | 0 | 0.01±0.00 |
| Thermoleophilia | 0 | 0.004±0.00 | 0 | 0 | 0 | 0 | 0 | 0.001±0.00 |
| Parcubacteria | 0 | 0.003±0.00 | 0 | 0 | 0 | 0 | 0 | 0 |

**Table S5.** Abundance of order level bacterial taxa in the GI sections and Feces (mean±SEM, % of assigned 16S rRNA gene sequences) Taxa above 1% abundance are written in a bold format.

| **Order** | **Stomach** | **Duodenum** | **Jejunum** | **Ileum** | **Cecum** | **Colon** | **Feces** | **GI** |
| --- | --- | --- | --- | --- | --- | --- | --- | --- |
| Bacteroidales | 35.21±9.84 | 42.76±12.9 | 55.17±18.88 | 56.8±20.43 | 49.27±2.46 | 58.41±9.37 | 61.66±4.49 | 49.6±3.39 |
| Lactobacillales | 23.12±15.4 | 17.2±10.46 | 13.71±7.8 | 19.51±14.31 | 0.16±0.09 | 0.12±0.08 | 0.11±0.07 | 12.3±3.69 |
| Rickettsiales | 1.14±0.68 | 3.86±1.58 | 2.49±2.14 | 3.41±2.07 | 0 | 0 | 0 | 1.82±0.42 |
| Enterobacteriales | 3.23±1.64 | 2.25±1.14 | 2.32±1.06 | 1.21±1.04 | 0 | 0.02±0 | 0 | 1.5±0.45 |
| Erysipelotrichales | 0.64±0.45 | 5.77±1.2 | 15.18±5.79 | 8.07±3.24 | 2.3±1.46 | 1.00±0.26 | 0.41±0.12 | 5.49±2.07 |
| Betaproteobacteriales | 0.71±0.47 | 2.5±0.7 | 3.37±2.16 | 3.05±1.05 | 1.3±1.09 | 0.85±0.35 | 1.63±1.02 | 1.97±0.43 |
| Clostridiales | 23.8±12.1 | 12.89±6.24 | 3.04±1.2 | 1.27±0.41 | 38.74±5.04 | 32.7±8.29 | 28.57±3.82 | 18.74±5.79 |
| Desulfovibrionales | 2.23±0.76 | 1.48±0.42 | 0.7±0.25 | 0.34±0.08 | 2.76±1.05 | 2.51±0.63 | 2.51±1.52 | 1.67±0.37 |
| Campylobacterales | 1.42±0.26 | 0.68±0 | 0 | 0.01±0 | 3.46±1.55 | 1.75±0.36 | 1.5±0.62 | 1.22±0.48 |
| Anaeroplasmatales | 0.13±0 | 0.1±0 | 0 | 0 | 0.46±0.25 | 0.72±0.1 | 1.73±1.29 | 0.24±0.1 |
| Chloroplast | 0.85±0 | 1.45±0.52 | 0.34±0 | 2.25±0 | 0 | 0 | 0 | 0.81±0.29 |
| Pasteurellales | 1.38±0 | 1.55±0 | 0.07±0 | 0 | 0 | 0 | 0 | 0.5±0.27 |
| Pseudomonadales | 1.01±0.86 | 1.51±1 | 0.01±0 | 0.28±0.21 | 0 | 0 | 0 | 0.47±0.24 |
| Saccharimonadales | 0.71±0.32 | 1.6±0.87 | 0.02±0 | 0.04±0 | 0.02±0 | 0.59±0.45 | 0.51±0 | 0.5±0.23 |
| Bifidobacteriales | 0.17±0 | 0.06±0 | 0.66±0.1 | 0.49±0.3 | 0.01±0 | 0 | 0 | 0.23±0.1 |
| Deferribacterales | 0.09±0 | 0.13±0 | 0 | 0 | 0.26±0.14 | 0.34±0.25 | 0.4±0 | 0.14±0.04 |
| Coriobacteriales | 0.23±0.16 | 0.41±0.16 | 0.44±0.33 | 0.38±0.31 | 0.13±0.1 | 0.02±0 | 0.21±0 | 0.27±0.06 |
| Bacillales | 0.88±0.27 | 0.44±0.23 | 0.47±0.37 | 0.28±0 | 0.01±0 | 0 | 0 | 0.35±0.12 |
| Mollicutes RF39 | 0.04±0 | 0.05±0.04 | 0 | 0.03±0 | 0.44±0.35 | 0.04±0.01 | 0.17±0.13 | 0.1±0.07 |
| Rhizobiales | 0.19±0.13 | 0.76±0.48 | 0.44±0.34 | 0.56±0.42 | 0 | 0 | 0 | 0.32±0.09 |
| Xanthomonadales | 0.08±0.02 | 0.43±0.06 | 0.85±0 | 0.24±0 | 0 | 0 | 0 | 0.27±0.12 |
| Planctomycetales | 0.3±0 | 0 | 0 | 0 | 0 | 0 | 0 | 0.05±0 |
| Mycoplasmatales | 0.03±0 | 0.16±0.06 | 0.01±0 | 0.18±0 | 0.03±0 | 0 | 0 | 0.07±0.03 |
| Flavobacteriales | 0.65±0.52 | 0.12±0 | 0 | 0.15±0 | 0 | 0 | 0 | 0.15±0.1 |
| Verrucomicrobiales | 0 | 0.18±0.15 | 0.1±0.01 | 0.1±0 | 0.07±0.02 | 0.04±0.02 | 0.01±0 | 0.08±0.02 |
| Selenomonadales | 0.05±0 | 0.11±0 | 0.12±0 | 0.25±0 | 0 | 0 | 0 | 0.09±0.03 |
| Nostocales | 0.71±0 | 0 | 0 | 0 | 0 | 0 | 0 | 0.12±0 |
| Rhodospirillales | 0.07±0.03 | 0.06±0.05 | 0.02±0 | 0 | 0.13±0.05 | 0.45±0.27 | 0.37±0.12 | 0.12±0.06 |
| Sphingomonadales | 0.16±0.11 | 0.44±0.16 | 0.07±0 | 0.22±0.16 | 0 | 0 | 0 | 0.15±0.06 |
| Micrococcales | 0.07±0 | 0.3±0.11 | 0.06±0.04 | 0.17±0 | 0 | 0 | 0 | 0.1±0.04 |
| Fusobacteriales | 0 | 0.06±0 | 0.04±0 | 0.14±0 | 0 | 0 | 0 | 0.04±0.02 |
| Gastranaerophilales | 0.18±0.01 | 0.06±0 | 0 | 0 | 0.21±0.01 | 0.39±0.16 | 0.19±0.01 | 0.14±0.05 |
| Frankiales | 0.18±0 | 0 | 0.06±0 | 0.14±0 | 0 | 0 | 0 | 0.06±0.02 |
| Opitutales | 0 | 0.12±0 | 0 | 0 | 0 | 0 | 0 | 0.02±0 |
| Gemmatimonadales | 0.08±0 | 0 | 0 | 0 | 0 | 0 | 0 | 0.01±0 |
| Chitinophagales | 0.01±0 | 0.1±0.02 | 0.02±0 | 0 | 0 | 0 | 0 | 0.02±0.02 |
| Pseudonocardiales | 0 | 0 | 0.07±0 | 0.04±0 | 0 | 0 | 0 | 0.02±0.01 |
| Alteromonadales | 0 | 0 | 0 | 0.08±0 | 0 | 0 | 0 | 0.01±0 |
| Gammaproteobacteria | 0 | 0.04±0 | 0 | 0 | 0 | 0 | 0 | 0.01±0 |
| Aeromonadales | 0 | 0.02±0 | 0 | 0.03±0 | 0 | 0 | 0 | 0.01±0 |
| Absconditabacteriales | 0 | 0 | 0.05±0 | 0 | 0 | 0 | 0 | 0.01±0 |
| Oceanospirillales | 0 | 0.04±0 | 0 | 0 | 0 | 0 | 0 | 0.01±0 |
| Corynebacteriales | 0.05±0 | 0.05±0.02 | 0 | 0 | 0 | 0 | 0 | 0.02±0 |
| Actinomycetales | 0 | 0.05±0 | 0 | 0 | 0 | 0 | 0 | 0.01±0 |
| Methylococcales | 0 | 0 | 0 | 0.04±0 | 0 | 0 | 0 | 0.01±0 |
| Caulobacterales | 0.01±0 | 0.06±0 | 0.01±0 | 0.07±0 | 0 | 0 | 0 | 0.02±0.01 |
| Cytophagales | 0.05±0 | 0 | 0 | 0 | 0 | 0 | 0 | 0.01±0 |
| Sphingobacteriales | 0.01±0 | 0.04±0 | 0 | 0.04±0 | 0 | 0 | 0 | 0.02±0 |
| MBNT15 | 0 | 0.04±0 | 0 | 0 | 0 | 0 | 0 | 0.01±0 |
| Acetobacterales | 0 | 0.02±0 | 0.04±0 | 0.02±0 | 0 | 0 | 0 | 0.01±0 |
| Vibrionales | 0.04±0 | 0 | 0 | 0 | 0 | 0 | 0 | 0.01±0 |
| Salinisphaerales | 0 | 0 | 0 | 0.02±0 | 0 | 0 | 0 | 0 |
| PLTA13 | 0 | 0.01±0 | 0 | 0 | 0 | 0 | 0 | 0 |
| Bdellovibrionales | 0 | 0 | 0.01±0 | 0 | 0 | 0 | 0 | 0 |

**Table S6**. Abundance of family level bacterial taxa in the GI sections and Feces (mean±SEM, % of assigned 16S rRNA gene sequences) Taxa above 1% abundance are written in a bold format.

| **Family** | **Stomach** | **Duodenum** | **Jejunum** | **Ileum** | **Cecum** | **Colon** | **Feces** | **GI** |
| --- | --- | --- | --- | --- | --- | --- | --- | --- |
| Muribaculaceae | 32.68±9.83 | 37.47±14.53 | 54.98±18.99 | 55.53±21.43 | 41.12±4.54 | 46.31±7.14 | 49.98±2.83 | 44.68±3.48 |
| Lactobacillaceae | 23.05±15.42 | 15.6±9.59 | 10.32±5.15 | 12.89±8.93 | 0.16±0.09 | 0.11±0.09 | 0.11±0.07 | 10.36±3.35 |
| Mitochondria | 1.14±0.68 | 3.86±1.58 | 2.49±2.14 | 3.41±2.07 | 0 | 0 | 0 | 1.82±0.42 |
| Streptococcaceae | 0.04±0 | 1.35±0.63 | 2.74±2.17 | 5.29±4.56 | 0 | 0 | 0 | 1.57±0.81 |
| Enterobacteriaceae | 3.23±1.64 | 2.25±1.14 | 2.32±1.06 | 1.21±1.04 | 0 | 0.02±0 | 0 | 1.5±0.45 |
| Erysipelotrichaceae | 0.64±0.45 | 5.77±1.2 | 15.18±5.79 | 8.07±3.24 | 2.3±1.46 | 1±0.26 | 0.41±0.12 | 5.49±2.07 |
| Burkholderiaceae | 0.71±0.47 | 2.5±0.7 | 3.31±2.11 | 3.05±1.05 | 1.18±1.00 | 0.84±0.35 | 1.58±0.97 | 1.93±0.43 |
| Clostridiales vadinBB60 group | 0.16±0.09 | 0.97±0 | 0 | 0 | 3.6±1.46 | 3.02±1.43 | 4.43±1.98 | 1.29±0.58 |
| Lachnospiraceae | 12.32±6.19 | 6.66±3.5 | 0.33±0.21 | 0.61±0.33 | 19.93±1.89 | 18.27±6.25 | 10.68±5.49 | 9.69±3.18 |
| Ruminococcaceae | 10.38±5.83 | 4.52±1.47 | 0.12±0 | 0.3±0.21 | 13.73±2.13 | 10.56±3.07 | 12.75±2.43 | 6.6±2.15 |
| Desulfovibrionaceae | 2.23±0.76 | 1.48±0.42 | 0.7±0.25 | 0.34±0.08 | 2.76±1.05 | 2.51±0.63 | 2.51±1.52 | 1.67±0.37 |
| Prevotellaceae | 0.83±0.66 | 2.2±0.3 | 0.07±0 | 0.41±0 | 2.75±1.39 | 4.69±2.6 | 4.56±2.31 | 1.82±0.65 |
| Helicobacteraceae | 1.34±0.3 | 0.68±0 | 0 | 0.01±0 | 3.46±1.55 | 1.75±0.36 | 1.5±0.62 | 1.21±0.48 |
| Bacteroidaceae | 0.62±0.03 | 1.04±0.33 | 0.09±0.07 | 0.71±0 | 1.54±0.31 | 1.94±0.34 | 2.15±0.88 | 0.99±0.25 |
| Rikenellaceae | 0.26±0.09 | 1.09±0.82 | 0 | 0 | 1.59±0.87 | 2.24±1.08 | 2.13±0.81 | 0.86±0.34 |
| Marinifilaceae | 0.07±0 | 0.57±0 | 0 | 0.05±0.04 | 1.62±0.56 | 1.2±0.86 | 0.88±0.56 | 0.59±0.25 |
| Chloroplast | 0.16±0 | 1.33±0.43 | 0.29±0 | 2.06±0 | 0 | 0 | 0 | 0.64±0.32 |
| Saccharimonadaceae | 0.71±0.32 | 1.57±0.86 | 0.02±0 | 0.04±0 | 0.02±0 | 0.59±0.45 | 0.51±0 | 0.49±0.23 |
| Pasteurellaceae | 1.38±0 | 1.55±0 | 0.07±0 | 0 | 0 | 0 | 0 | 0.5±0.27 |
| Moraxellaceae | 1.01±0.86 | 1.48±1.01 | 0.01±0 | 0.11±0.07 | 0 | 0 | 0 | 0.44±0.25 |
| Clostridiaceae | 0.48±0.4 | 0.28±0 | 1.29±0.48 | 0.06±0.04 | 0.01±0 | 0 | 0 | 0.35±0.19 |
| Peptostreptococcaceae | 0.09±0 | 0.28±0.23 | 1.28±0.67 | 0.28±0.11 | 0.03±0.02 | 0 | 0 | 0.33±0.19 |
| Tannerellaceae | 0.47±0.31 | 0.35±0.3 | 0 | 0.09±0 | 0.22±0.1 | 1.28±0.56 | 1.03±0.6 | 0.4±0.17 |
| Anaeroplasmataceae | 0.13±0 | 0.1±0 | 0 | 0 | 0.46±0.25 | 0.72±0.1 | 1.73±1.29 | 0.24±0.1 |
| Bacteroidales | 0.25±0.21 | 0.02±0 | 0.03±0 | 0 | 0.44±0.06 | 0.75±0.29 | 0.94±0.41 | 0.25±0.11 |
| Enterococcaceae | 0.03±0.01 | 0.24±0.15 | 0.65±0 | 1.32±0 | 0 | 0 | 0 | 0.37±0.2 |
| Clostridiales | 0.24±0 | 0.02±0 | 0.01±0 | 0 | 0.98±0.29 | 0.62±0.23 | 0.32±0.11 | 0.31±0.15 |
| Xanthomonadaceae | 0.08±0.02 | 0.43±0.06 | 0.85±0 | 0.18±0 | 0 | 0 | 0 | 0.26±0.12 |
| Bifidobacteriaceae | 0.17±0 | 0.06±0 | 0.66±0.1 | 0.49±0.3 | 0.01±0 | 0 | 0 | 0.23±0.1 |
| Family XI | 0.77±0.2 | 0 | 0.42±0.36 | 0.16±0 | 0.01±0 | 0 | 0 | 0.23±0.12 |
| Rhizobiaceae | 0.18±0.13 | 0.42±0.23 | 0.29±0.22 | 0.46±0.36 | 0 | 0 | 0 | 0.22±0.05 |
| Atopobiaceae | 0 | 0.21±0.16 | 0.42±0.36 | 0.36±0 | 0.1±0 | 0.02±0 | 0.21±0 | 0.19±0.06 |
| Deferribacteraceae | 0.09±0 | 0.13±0 | 0 | 0 | 0.26±0.14 | 0.34±0.25 | 0.4±0 | 0.14±0.04 |
| uncultured | 0.07±0.03 | 0.06±0.05 | 0.02±0 | 0 | 0.13±0.05 | 0.45±0.27 | 0.37±0.12 | 0.12±0.06 |
| Weeksellaceae | 0.65±0.52 | 0.09±0 | 0 | 0.15±0 | 0 | 0 | 0 | 0.15±0.1 |
| Sphingomonadaceae | 0.16±0.11 | 0.44±0.16 | 0.07±0 | 0.22±0.16 | 0 | 0 | 0 | 0.15±0.06 |
| Family XIII | 0.09±0.04 | 0.03±0.02 | 0.01±0 | 0 | 0.24±0.14 | 0.05±0.04 | 0.29±0.14 | 0.07±0.03 |
| Coleofasciculaceae | 0.71±0 | 0 | 0 | 0 | 0 | 0 | 0 | 0.12±0 |
| Phaseolus acutifolius (tepary bean) | 0.68±0 | 0 | 0 | 0 | 0 | 0 | 0 | 0.11±0 |
| Gastranaerophilales | 0.09±0 | 0 | 0 | 0 | 0.1±0.05 | 0.28±0.05 | 0.05±0.01 | 0.08±0.04 |
| Akkermansiaceae | 0 | 0.18±0.15 | 0.1±0.01 | 0.1±0 | 0.07±0.02 | 0.04±0.02 | 0.01±0 | 0.08±0.02 |
| Veillonellaceae | 0.03±0 | 0.11±0 | 0.12±0 | 0.25±0 | 0 | 0 | 0 | 0.08±0.03 |
| Xanthobacteraceae | 0 | 0.32±0.27 | 0.07±0 | 0.09±0.05 | 0 | 0 | 0 | 0.08±0.05 |
| Brevibacteriaceae | 0 | 0.23±0.04 | 0.06±0.04 | 0.17±0 | 0 | 0 | 0 | 0.08±0.03 |
| Firmicutes bacterium CAG:822 | 0 | 0 | 0 | 0 | 0.37±0 | 0 | 0.06±0 | 0.06±0.08 |
| Mycoplasmataceae | 0.03±0 | 0.16±0.06 | 0.01±0 | 0.18±0 | 0.03±0 | 0 | 0 | 0.07±0.03 |
| Bacillaceae | 0.01±0 | 0.29±0.14 | 0.03±0 | 0.09±0 | 0 | 0 | 0 | 0.07±0.04 |
| Peptococcaceae | 0.04±0 | 0.07±0 | 0 | 0 | 0.13±0.06 | 0.12±0.09 | 0.01±0 | 0.06±0.01 |
| Eggerthellaceae | 0.13±0.08 | 0.16±0.05 | 0.02±0.01 | 0.01±0 | 0.02±0 | 0 | 0 | 0.06±0.03 |
| Ambiguous_taxa | 0.08±0 | 0.06±0 | 0 | 0 | 0.07±0 | 0.07±0.04 | 0.03±0 | 0.05±0 |
| uncultured | 0.3±0 | 0 | 0 | 0 | 0 | 0 | 0 | 0.05±0 |
| Betaproteobacteriales | 0 | 0 | 0.06±0 | 0.01±0 | 0.13±0.09 | 0.01±0 | 0.05±0 | 0.03±0.02 |
| Fusobacteriaceae | 0 | 0.06±0 | 0.04±0 | 0.14±0 | 0 | 0 | 0 | 0.04±0.02 |
| Geodermatophilaceae | 0.18±0 | 0 | 0 | 0.03±0 | 0 | 0 | 0 | 0.03±0.03 |
| Mollicutes RF39 | 0.01±0 | 0.02±0.01 | 0 | 0.03±0 | 0.04±0.03 | 0.04±0.01 | 0.07±0 | 0.02±0.01 |
| Pseudomonadaceae | 0 | 0.03±0 | 0 | 0.17±0 | 0 | 0 | 0 | 0.03±0.03 |
| Triticum aestivum (bread wheat) | 0 | 0.04±0 | 0.05±0 | 0.1±0 | 0 | 0 | 0 | 0.03±0.01 |
| Paenibacillaceae | 0 | 0.14±0.05 | 0.02±0 | 0.04±0 | 0 | 0 | 0 | 0.03±0.02 |
| Frankiales | 0 | 0 | 0.06±0 | 0.12±0 | 0 | 0 | 0 | 0.03±0.01 |
| Christensenellaceae | 0 | 0.06±0 | 0 | 0.02±0 | 0.04±0.02 | 0 | 0.04±0.02 | 0.02±0.01 |
| Chitinophagaceae | 0.01±0 | 0.1±0.02 | 0.02±0 | 0 | 0 | 0 | 0 | 0.02±0.02 |
| Caulobacteraceae | 0.01±0 | 0.06±0 | 0.01±0 | 0.07±0 | 0 | 0 | 0 | 0.02±0.01 |
| Defluviitaleaceae | 0 | 0 | 0 | 0 | 0.05±0 | 0.04±0.03 | 0.04±0 | 0.02±0.01 |
| Staphylococcaceae | 0.1±0 | 0.02±0 | 0.01±0 | 0 | 0 | 0 | 0 | 0.02±0.02 |
| uncultured bacterium | 0.03±0.01 | 0.03±0 | 0 | 0 | 0.02±0 | 0 | 0.03±0 | 0.01±0 |
| Puniceicoccaceae | 0 | 0.12±0 | 0 | 0 | 0 | 0 | 0 | 0.02±0 |
| Clostridium sp. K4410.MGS-306 | 0.01±0 | 0 | 0 | 0 | 0.03±0 | 0 | 0.07±0.02 | 0.01±0.01 |
| Pseudonocardiaceae | 0 | 0 | 0.07±0 | 0.04±0 | 0 | 0 | 0 | 0.02±0.01 |
| Microbacteriaceae | 0.07±0 | 0.04±0 | 0 | 0 | 0 | 0 | 0 | 0.02±0 |
| Beijerinckiaceae | 0 | 0.03±0 | 0.08±0 | 0.01±0 | 0 | 0 | 0 | 0.02±0.01 |
| Sphingobacteriaceae | 0.01±0 | 0.04±0 | 0 | 0.04±0 | 0 | 0 | 0 | 0.02±0 |
| Triticum urartu | 0 | 0.06±0 | 0 | 0.03±0 | 0 | 0 | 0 | 0.01±0.01 |
| Gemmatimonadaceae | 0.08±0 | 0 | 0 | 0 | 0 | 0 | 0 | 0.01±0 |
| Coriobacteriales | 0.04±0 | 0.04±0 | 0 | 0.01±0 | 0 | 0 | 0 | 0.01±0.01 |
| Acetobacteraceae | 0 | 0.02±0 | 0.04±0 | 0.02±0 | 0 | 0 | 0 | 0.01±0 |
| Shewanellaceae | 0 | 0 | 0 | 0.08±0 | 0 | 0 | 0 | 0.01±0 |
| Corynebacteriaceae | 0.05±0 | 0.03±0 | 0 | 0 | 0 | 0 | 0 | 0.01±0 |
| Campylobacteraceae | 0.07±0.05 | 0 | 0 | 0 | 0 | 0 | 0 | 0.01±0 |
| Aegilops tauschii | 0 | 0 | 0 | 0.07±0 | 0 | 0 | 0 | 0.01±0 |
| Coriobacteriaceae | 0.06±0 | 0 | 0 | 0 | 0 | 0 | 0 | 0.01±0 |
| <0.01 | 0.17±0.08 | 0.33±0.09 | 0.06±0.05 | 0.16±0.13 | 0.01±0 | 0.03±0.01 | 0.07±0.01 | 0.13±0.04 |

**Table S7**. Abundance of genus level bacterial taxa in the GI sections and Feces (mean±SEM, % of assigned 16S rRNA gene sequences) Taxa above 1% abundance are written in a bold format.

| **Genus** | **Stomach** | **Duodenum** | **Jejunum** | **Ileum** | **Cecum** | **Colon** | **Feces** | **GI** |
| --- | --- | --- | --- | --- | --- | --- | --- | --- |
| uncultured *Muribaculaceae* | 18.25±6.04 | 24.03±10.36 | 24.72±8.91 | 27.5±10.99 | 30.1±2.33 | 31.91±4.72 | 35.66±2.7 | 26.09±1.99 |
| *Ambiguous_taxa* | 12.17±5.51 | 8.35±3.28 | 23.32±9.03 | 20.77±8.97 | 8.51±0.95 | 9.53±2.26 | 9.79±1.06 | 13.77±2.7 |
| uncultured *Bacteroidales bacterium* | 3.00±2.00 | 2.73±0.46 | 2.72±0.23 | 3.76±1.09 | 4.6±0.37 | 2.63±0.52 | 2.97±0.15 | 3.24±0.32 |
| *Muribaculum* | 1.46±0.43 | 1.03±0.47 | 3.78±1.53 | 1.62±1.25 | 1.12±0.43 | 2.65±0.61 | 1.91±0.57 | 1.94±0.44 |
| *Lactobacillus* | 25.26±16.67 | 18.23±11.24 | 11.68±5.87 | 15.73±11.01 | 0.19±0.1 | 0.13±0.1 | 0.13±0.08 | 11.87±4.12 |
| *Lactococcus* | 0 | 1.28±0.64 | 2.55±2.11 | 6.3±0 | 0 | 0 | 0 | 1.69±1.07 |
| *Dubosiella* | 0.37±0.31 | 1.48±0.27 | 11.36±6.59 | 5.43±2.06 | 1.59±1.37 | 0.76±0.39 | 0.28±0.13 | 3.5±1.74 |
| *Parasutterella* | 0.69±0.53 | 2.18±0.69 | 3.52±2.28 | 2.94±1.13 | 1.21±1.02 | 0.98±0.13 | 1.78±1.09 | 1.92±0.47 |
| *Turicibacter* | 0.29±0 | 3.2±0.91 | 2.54±0.33 | 1.73±0.42 | 0.38±0.11 | 0.27±0.19 | 0.02±0.01 | 1.4±0.52 |
| *Lachnospiraceae NK4A136 group* | 2.98±1.34 | 1.53±1.31 | 0.19±0 | 0 | 6.67±1.2 | 8.35±3.8 | 5.29±3.92 | 3.29±1.41 |
| *Ruminococcaceae UCG-014* | 1.85±0.82 | 1.22±0.57 | 0 | 0.01±0 | 2.65±1.11 | 2.63±0.72 | 4.82±2.55 | 1.39±0.45 |
| *uncultured Lachnospiraceae* | 1.56±0.54 | 1.31±0.38 | 0 | 0.13±0.1 | 2.55±0.85 | 2.55±1.63 | 0.68±0.25 | 1.35±0.41 |
| *Helicobacter* | 1.62±0.42 | 0.86±0 | 0 | 0.01±0 | 4.15±1.79 | 2.05±0.44 | 1.72±0.71 | 1.45±0.64 |
| uncultured *Ruminococcaceae* | 1.87±1.2 | 0.94±0.68 | 0 | 0 | 2.44±0.71 | 1.71±1.24 | 1.22±0.42 | 1.16±0.39 |
| *Ruminiclostridium 9* | 1.21±1.01 | 0.23±0 | 0 | 0 | 1.48±0.48 | 2.06±0.41 | 2.01±0.4 | 0.83±0.31 |
| uncultured *Desulfovibrionaceae* | 1.17±0.74 | 0.93±0.78 | 0 | 0 | 2.73±1.17 | 2.62±0.6 | 2.26±1.34 | 1.24±0.39 |
| *Bacteroides* | 0.74±0.06 | 1.28±0.44 | 0.1±0.08 | 0.87±0 | 1.87±0.33 | 2.26±0.39 | 2.49±1.03 | 1.18±0.32 |
| *Alloprevotella* | 0.58±0 | 1.11±0 | 0 | 0 | 1.65±0.84 | 3.31±2.02 | 2.6±1.56 | 1.11±0.48 |
| uncultured *Clostridiales vadinBB60 group* | 0.09±0.06 | 0.71±0 | 0 | 0 | 2.25±0.92 | 1.47±0.53 | 3.07±1.37 | 0.75±0.38 |
| *Odoribacter* | 0.1±0 | 0.71±0 | 0 | 0 | 1.98±0.59 | 1.39±0.99 | 1.01±0.65 | 0.7±0.35 |
| *Alistipes* | 0.14±0.06 | 1±0.75 | 0 | 0 | 1.93±1.11 | 1.63±0.99 | 1.94±1.04 | 0.78±0.32 |
| *Morganella* | 1.23±0.37 | 1.82±0 | 1.56±0 | 0.63±0.55 | 0 | 0 | 0 | 0.88±0.21 |
| *Clostridium* | 1.17±0 | 1.78±1.24 | 0 | 0.07±0.06 | 0 | 0 | 0 | 0.5±0.35 |
| *Angelakisella* | 1.26±1.08 | 0.2±0.09 | 0 | 0 | 1.26±0.05 | 0.38±0.28 | 0.96±0.37 | 0.52±0.23 |
| *Desulfovibrio* | 1.25±0.49 | 0.8±0.23 | 0.79±0.29 | 0.39±0.09 | 0.23±0.09 | 0.15±0.09 | 0.09±0 | 0.6±0.17 |
| *Rodentibacter* | 1.08±0 | 0 | 0.04±0 | 0 | 0 | 0 | 0 | 0.19±0.3 |
| *Candidatus Saccharimonas* | 0.89±0.43 | 1.76±0.9 | 0 | 0.05±0 | 0.02±0 | 0.68±0.53 | 0.59±0 | 0.57±0.29 |
| *Ileibacterium* | 0.01±0 | 0.63±0.44 | 0.5±0.4 | 1.12±0.96 | 0.05±0 | 0.01±0 | 0.01±0 | 0.39±0.18 |
| *Allobaculum* | 0.05±0 | 0.96±0.17 | 2.53±0.46 | 0.9±0.48 | 0.16±0.13 | 0.08±0.03 | 0.1±0 | 0.78±0.39 |
| *Parabacteroides* | 0.6±0.41 | 0.44±0.37 | 0 | 0.12±0 | 0.29±0.14 | 1.46±0.63 | 1.18±0.68 | 0.48±0.21 |
| *Tyzzerella* | 0.02±0 | 0.29±0 | 0 | 0 | 1.2±0.56 | 1.5±1.04 | 0.94±0.79 | 0.5±0.29 |
| *Ruminiclostridium* | 0.94±0.4 | 0.53±0.45 | 0 | 0 | 1.1±0.41 | 0.77±0.31 | 0.43±0.21 | 0.56±0.17 |
| *Anaeroplasma* | 0.17±0 | 0.12±0 | 0 | 0 | 0.6±0.34 | 0.84±0.11 | 1.97±1.46 | 0.29±0.14 |
| *Eubacterium xylanophilum group* | 0.77±0 | 0.05±0.01 | 0 | 0 | 0.73±0.19 | 0.48±0.08 | 1.13±0.2 | 0.34±0.14 |
| *Intestinimonas* | 0.35±0.24 | 0.01±0 | 0 | 0 | 0.58±0.32 | 0.77±0.39 | 0.96±0.43 | 0.29±0.13 |
| *Enterococcus* | 0.04±0.02 | 0.28±0.18 | 0.67±0 | 1.62±0 | 0 | 0 | 0 | 0.43±0.28 |
| *Lachnoclostridium* | 0.47±0.36 | 0.54±0.1 | 0.04±0 | 0.09±0 | 0.9±0.07 | 0.41±0.11 | 0.1±0 | 0.41±0.13 |
| *Ruminiclostridium 5* | 0.77±0.55 | 0.5±0.29 | 0 | 0.01±0 | 0.59±0.24 | 0.31±0.01 | 0.35±0.11 | 0.36±0.12 |
| *uncultured organism Muribaculaceae* | 0.82±0.33 | 0.41±0.05 | 0.04±0 | 0.07±0 | 0.21±0 | 0.4±0 | 0.5±0.37 | 0.33±0.12 |
| *ASF356* | 0.58±0.36 | 0.09±0 | 0 | 0 | 1.2±0.81 | 0.2±0 | 0.31±0.13 | 0.35±0.21 |
| *Prevotellaceae NK3B31 group* | 0 | 0.18±0 | 0 | 0 | 0.77±0.38 | 0.52±0 | 0.79±0.34 | 0.25±0.12 |
| *Muribacter* | 0.4±0 | 1.84±0 | 0 | 0 | 0 | 0 | 0 | 0.37±0.41 |
| *mouse gut metagenome* | 0.09±0.07 | 0.27±0.2 | 0.11±0.02 | 0.79±0 | 0.02±0 | 0.51±0.37 | 0.31±0.02 | 0.3±0.12 |
| *Escherichia-Shigella* | 0 | 0.44±0 | 0.83±0.54 | 0.71±0.61 | 0 | 0 | 0 | 0.33±0.15 |
| *Eubacterium oxidoreducens group* | 0.4±0.34 | 0.01±0 | 0 | 0 | 0.58±0.27 | 0.38±0.13 | 0.47±0.2 | 0.23±0.1 |
| *Prevotellaceae Ga6A1 group* | 0.01±0 | 0 | 0 | 0 | 0.23±0.09 | 0.62±0.41 | 0.91±0.4 | 0.15±0.12 |
| *Roseburia* | 0.04±0 | 0 | 0 | 0 | 0.45±0.02 | 0.81±0.26 | 0.4±0 | 0.22±0.15 |
| *Firmicutes bacterium CAG:822* | 0.57±0.46 | 0.35±0 | 0.71±0.6 | 0.06±0.04 | 0 | 0 | 0 | 0.28±0.11 |
| *Prevotellaceae UCG-001* | 0.37±0 | 0.22±0 | 0 | 0 | 0.17±0.11 | 0.6±0.51 | 0.25±0.09 | 0.23±0.08 |
| *uncultured Clostridia bacterium* | 0.02±0 | 0.2±0 | 0 | 0 | 0.61±0.26 | 0.21±0.09 | 0.56±0.48 | 0.17±0.1 |
| *Ruminococcus 1* | 0.9±0.23 | 0 | 0.48±0.4 | 0.19±0 | 0.01±0 | 0 | 0 | 0.26±0.15 |
| *Bifidobacterium* | 0.19±0 | 0.07±0 | 0.74±0.13 | 0.57±0.34 | 0.01±0 | 0 | 0 | 0.26±0.13 |
| *Gemella* | 0.24±0.12 | 0 | 0 | 0 | 0.53±0.07 | 0.71±0.43 | 0.02±0 | 0.25±0.13 |
| *Mucispirillum* | 0.12±0 | 0.16±0 | 0 | 0 | 0.31±0.16 | 0.39±0.29 | 0.46±0 | 0.16±0.05 |
| *Coriobacteriaceae UCG-002* | 0 | 0.23±0 | 0.47±0.4 | 0.45±0 | 0 | 0 | 0.24±0 | 0.19±0.09 |
| *Ambiguous_taxa* | 0.18±0 | 0 | 0 | 0 | 0.05±0.01 | 0.6±0.35 | 0.44±0.35 | 0.14±0.12 |
| *Lachnospiraceae UCG-001* | 0.35±0.22 | 0.01±0 | 0 | 0 | 0.1±0 | 0.7±0.5 | 0.07±0 | 0.19±0.13 |
| *Streptococcus* | 0.05±0 | 0.33±0.07 | 0.56±0.47 | 0.2±0.15 | 0 | 0 | 0 | 0.19±0.09 |
| *Stenotrophomonas* | 0.09±0.02 | 0.34±0.11 | 0.69±0 | 0 | 0 | 0 | 0 | 0.19±0.12 |
| *Prevotella 9* | 0 | 0.76±0 | 0 | 0.33±0 | 0 | 0 | 0 | 0.18±0.12 |
| *Acinetobacter* | 0.62±0.22 | 0.11±0 | 0 | 0.01±0 | 0.09±0 | 0 | 0.14±0 | 0.14±0.11 |
| *Sphingomonas* | 0.17±0.12 | 0.46±0.12 | 0.08±0 | 0.26±0.2 | 0 | 0 | 0 | 0.16±0.07 |
| *Ruminococcaceae UCG-010* | 0.18±0 | 0.13±0.03 | 0 | 0 | 0.18±0.04 | 0.23±0.16 | 0.24±0.1 | 0.12±0.04 |
| *Eubacterium ruminantium group* | 0.42±0 | 0 | 0 | 0.04±0 | 0.24±0.19 | 0.25±0.16 | 0 | 0.16±0.06 |
| *Rikenella* | 0.02±0 | 0.01±0 | 0 | 0.01±0 | 0.02±0 | 0.47±0.24 | 0.41±0.16 | 0.09±0.08 |
| *Eubacterium coprostanoligenes group* | 0.02±0 | 0.01±0 | 0 | 0.08±0 | 0.41±0 | 0.19±0.13 | 0.22±0 | 0.12±0.07 |
| *Rikenellaceae RC9 gut group* | 0 | 0.29±0 | 0 | 0 | 0.1±0.06 | 0.46±0.28 | 0.07±0.02 | 0.14±0.08 |
| *Faecalibaculum* | 0.03±0 | 0.39±0 | 0.09±0.06 | 0 | 0.38±0 | 0.01±0 | 0.01±0 | 0.15±0.08 |
| *Acetatifactor* | 0.02±0 | 0.15±0 | 0 | 0 | 0.48±0.38 | 0.18±0 | 0.06±0 | 0.14±0.08 |
| *Ralstonia* | 0.08±0 | 0.4±0.34 | 0.01±0 | 0.33±0 | 0 | 0 | 0 | 0.13±0.08 |
| *Phaseolus acutifolius* (tepary bean) | 0.8±0 | 0 | 0 | 0 | 0 | 0 | 0 | 0.13±0 |
| *Microcoleus PCC-7113* | 0.77±0 | 0 | 0 | 0 | 0 | 0 | 0 | 0.13±0 |
| *Candidatus Arthromitus* | 0 | 0 | 0.75±0.58 | 0 | 0.01±0 | 0 | 0 | 0.13±0.21 |
| *Allorhizobium* | 0 | 0.32±0.14 | 0 | 0.42±0 | 0 | 0 | 0 | 0.12±0.03 |
| *Lachnospiraceae UCG-004* | 0.06±0 | 0 | 0 | 0 | 0.21±0.05 | 0.27±0.04 | 0.17±0.06 | 0.09±0.05 |
| *Bergeyella* | 0.69±0 | 0 | 0 | 0 | 0 | 0 | 0 | 0.12±0 |
| *Faecalibacterium* | 0 | 0.51±0.3 | 0.1±0 | 0.07±0 | 0 | 0 | 0 | 0.11±0.1 |
| *Ruminiclostridium 6* | 0.21±0.03 | 0.12±0 | 0 | 0 | 0.14±0.07 | 0.17±0 | 0 | 0.11±0.02 |
| *Fournierella* | 0.19±0.01 | 0 | 0 | 0 | 0.27±0.21 | 0.17±0 | 0 | 0.11±0.02 |
| *Blautia* | 0.09±0.06 | 0.21±0.14 | 0.01±0 | 0.08±0 | 0.03±0.01 | 0.09±0.04 | 0.09±0 | 0.09±0.03 |
| *Megamonas* | 0.03±0 | 0.12±0 | 0.12±0 | 0.31±0 | 0 | 0 | 0 | 0.1±0.05 |
| *Bilophila* | 0.09±0 | 0.19±0.04 | 0 | 0 | 0.23±0.06 | 0.07±0 | 0.01±0 | 0.1±0.03 |
| *Akkermansia* | 0.01±0 | 0.2±0.16 | 0.11±0.01 | 0.11±0 | 0.09±0.02 | 0.05±0.02 | 0.01±0 | 0.09±0.03 |
| *Ruminococcaceae UCG-009* | 0 | 0.02±0 | 0 | 0 | 0.42±0.15 | 0.09±0.03 | 0.02±0.01 | 0.09±0.08 |
| *Brevibacterium* | 0 | 0.28±0.04 | 0.07±0.05 | 0.2±0 | 0 | 0 | 0 | 0.09±0.04 |
| *Negativibacillus* | 0 | 0 | 0 | 0 | 0.27±0.15 | 0.04±0 | 0.24±0.11 | 0.05±0.07 |
| *A2* | 0.27±0 | 0.08±0 | 0 | 0 | 0.13±0.06 | 0.03±0.01 | 0.05±0 | 0.08±0.04 |
| *Oscillospira* | 0.23±0.19 | 0 | 0 | 0 | 0.18±0 | 0.13±0 | 0 | 0.09±0.02 |
| *Paraprevotella* | 0 | 0 | 0 | 0 | 0.44±0 | 0 | 0.07±0 | 0.07±0.13 |
| *Eubacterium fissicatena group* | 0 | 0 | 0 | 0 | 0.31±0.19 | 0.19±0.02 | 0 | 0.08±0.03 |
| *Mycoplasma* | 0.03±0 | 0.2±0.08 | 0.01±0 | 0.2±0 | 0.04±0 | 0 | 0 | 0.08±0.04 |
| *Proteus* | 0.29±0 | 0 | 0.2±0.14 | 0 | 0 | 0 | 0 | 0.08±0.02 |
| *GCA-900066575* | 0.09±0.01 | 0 | 0 | 0 | 0.15±0.06 | 0.16±0.07 | 0.07±0.03 | 0.07±0.02 |
| *Anaerovorax* | 0 | 0 | 0 | 0 | 0.19±0.07 | 0 | 0.27±0.11 | 0.03±0 |
| *Lysobacter* | 0 | 0.04±0 | 0.27±0 | 0.13±0 | 0 | 0 | 0 | 0.07±0.05 |
| *Oscillibacter* | 0 | 0 | 0 | 0.02±0 | 0 | 0.04±0 | 0.38±0 | 0.01±0 |
| *Prevotella 2* | 0 | 0.22±0 | 0.08±0 | 0.15±0 | 0 | 0 | 0 | 0.07±0.03 |
| *Azospirillum sp. 47_25* | 0.04±0.02 | 0 | 0 | 0 | 0.12±0.06 | 0.17±0.06 | 0.1±0.04 | 0.06±0.03 |
| *Ruminococcaceae UCG-013* | 0.11±0 | 0.21±0 | 0 | 0.01±0 | 0.04±0 | 0.02±0 | 0.02±0 | 0.07±0.03 |
| *Ruminococcaceae NK4A214 group* | 0 | 0.11±0 | 0 | 0 | 0.17±0.06 | 0.05±0.03 | 0.08±0.05 | 0.06±0.02 |
| *Lachnospiraceae FCS020 group* | 0.11±0 | 0.07±0 | 0 | 0 | 0.1±0 | 0.09±0.05 | 0.03±0 | 0.06±0.01 |
| uncultured  *Peptococcaceae* | 0.06±0 | 0.04±0 | 0 | 0 | 0.13±0.07 | 0.14±0.1 | 0.01±0 | 0.06±0.02 |
| *Agathobacter* | 0.09±0 | 0.24±0 | 0.04±0 | 0 | 0 | 0 | 0 | 0.06±0.04 |
| *Ruminococcaceae UCG-005* | 0.05±0 | 0.06±0 | 0 | 0 | 0.14±0.04 | 0.09±0.02 | 0.03±0.02 | 0.06±0.02 |
| *Ambiguous_taxa* | 0.06±0 | 0.11±0 | 0 | 0.18±0 | 0 | 0 | 0 | 0.06±0.02 |
| *Ambiguous_taxa* | 0.32±0 | 0 | 0 | 0 | 0 | 0 | 0 | 0.05±0 |
| *Millionella* | 0.01±0 | 0.04±0 | 0 | 0 | 0.12±0.03 | 0.1±0.07 | 0.03±0 | 0.05±0.02 |
| *Fusobacterium* | 0 | 0.07±0 | 0.05±0 | 0.17±0 | 0 | 0 | 0 | 0.05±0.03 |
| *Butyricicoccus* | 0.16±0.1 | 0.04±0.03 | 0 | 0 | 0.03±0.03 | 0.04±0.01 | 0.02±0 | 0.04±0.03 |
| *Collinsella* | 0.01±0 | 0.2±0.05 | 0 | 0.05±0 | 0 | 0 | 0 | 0.04±0.04 |
| *Ruminococcaceae UCG-002* | 0.07±0.04 | 0.05±0 | 0 | 0.09±0 | 0.04±0 | 0 | 0 | 0.04±0.01 |
| *Enterorhabdus* | 0.13±0.1 | 0.08±0.04 | 0.03±0.01 | 0.01±0 | 0.01±0 | 0 | 0 | 0.04±0.02 |
| *Oceanobacillus* | 0 | 0.15±0.1 | 0.04±0 | 0.06±0 | 0 | 0 | 0 | 0.04±0.02 |
| *Erysipelatoclostridium* | 0.01±0 | 0.01±0 | 0 | 0 | 0.13±0 | 0.04±0.03 | 0.06±0 | 0.03±0.02 |
| *Pseudomonas* | 0 | 0.04±0 | 0 | 0.21±0 | 0 | 0 | 0 | 0.04±0.05 |
| *Sutterella* | 0 | 0.06±0 | 0.04±0 | 0.14±0 | 0 | 0 | 0 | 0.04±0.02 |
| *Pseudoxanthomonas* | 0 | 0.15±0 | 0 | 0.09±0 | 0 | 0 | 0 | 0.04±0.02 |
| *Harryflintia* | 0.1±0 | 0.03±0 | 0 | 0 | 0.04±0 | 0.06±0 | 0 | 0.04±0.01 |
| *Triticum aestivum* (bread wheat) | 0 | 0.03±0 | 0.05±0 | 0.15±0 | 0 | 0 | 0 | 0.04±0.03 |
| *Triticum aestivum* (bread wheat) | 0 | 0.04±0 | 0.06±0 | 0.13±0 | 0 | 0 | 0 | 0.04±0.02 |
| *Paenibacillus* | 0 | 0.16±0.05 | 0.02±0 | 0.04±0 | 0 | 0 | 0 | 0.04±0.03 |
| *Papillibacter* | 0.04±0 | 0 | 0 | 0 | 0.09±0 | 0 | 0.09±0 | 0.02±0.01 |
| *Klebsiella* | 0 | 0.21±0.12 | 0 | 0 | 0 | 0 | 0 | 0.04±0 |
| uncultured bacterium *Rhodospirillales* | 0.03±0 | 0 | 0 | 0 | 0.03±0 | 0.09±0 | 0.06±0 | 0.02±0.01 |
| *Pygmaiobacter* | 0 | 0 | 0 | 0 | 0.07±0 | 0.07±0 | 0.05±0 | 0.02±0 |
| *Blastococcus* | 0.18±0 | 0 | 0 | 0 | 0 | 0 | 0 | 0.03±0 |
| *Chryseobacterium* | 0.06±0.01 | 0.06±0 | 0 | 0 | 0.01±0 | 0.04±0 | 0 | 0.03±0.01 |
| *Lachnospiraceae UCG-006* | 0.05±0 | 0.08±0.02 | 0 | 0 | 0.03±0 | 0.02±0 | 0 | 0.03±0.01 |
| uncultured *Chitinophagaceae* | 0.01±0 | 0.12±0.02 | 0.03±0 | 0.01±0 | 0 | 0 | 0 | 0.03±0.02 |
| *Christensenellaceae R-7 group* | 0 | 0.07±0 | 0 | 0.03±0 | 0.04±0 | 0 | 0.01±0 | 0.02±0.01 |
| *Defluviitaleaceae UCG-011* | 0 | 0 | 0 | 0 | 0.06±0 | 0.04±0 | 0.04±0 | 0.02±0.01 |
| uncultured *Puniceicoccaceae* | 0 | 0.15±0 | 0 | 0 | 0 | 0 | 0 | 0.02±0 |
| *Anaerostipes* | 0 | 0.09±0 | 0 | 0.05±0 | 0 | 0 | 0 | 0.02±0.01 |
| *Eubacterium eligens group* | 0 | 0.1±0 | 0.05±0.04 | 0 | 0 | 0 | 0 | 0.02±0.01 |
| *Staphylococcus* | 0.12±0 | 0.02±0 | 0.01±0 | 0 | 0 | 0 | 0 | 0.02±0.02 |
| uncultured bacterium *Mollicutes RF39* | 0.04±0 | 0.04±0 | 0 | 0 | 0.02±0 | 0 | 0.04±0 | 0.02±0 |
| *Enterobacter* | 0 | 0.14±0.04 | 0 | 0 | 0 | 0 | 0 | 0.02±0 |
| *Clostridium sp. K4410.MGS-306* | 0.01±0 | 0 | 0 | 0 | 0.04±0 | 0.01±0 | 0.08±0.02 | 0.01±0.01 |
| *Pseudonocardia* | 0 | 0 | 0.08±0 | 0.05±0 | 0 | 0 | 0 | 0.02±0.01 |
| *Prevotella 7* | 0 | 0.1±0 | 0 | 0 | 0 | 0 | 0.03±0 | 0.02±0 |
| *Eubacterium nodatum group* | 0.01±0 | 0 | 0 | 0 | 0.08±0 | 0.03±0 | 0 | 0.02±0.01 |
| *Lachnospiraceae NC2004 group* | 0.05±0 | 0 | 0 | 0 | 0.05±0.02 | 0.01±0.01 | 0 | 0.02±0.01 |
| *Bradyrhizobium* | 0 | 0.01±0 | 0 | 0.11±0.06 | 0 | 0 | 0 | 0.02±0.03 |
| *Comamonas* | 0 | 0.11±0.08 | 0 | 0 | 0 | 0 | 0 | 0.02±0 |
| *Sphingobacterium* | 0.02±0 | 0.04±0 | 0 | 0.05±0 | 0 | 0 | 0 | 0.02±0.01 |
| *Achromobacter* | 0 | 0.06±0.01 | 0 | 0.05±0 | 0 | 0 | 0 | 0.02±0 |
| *Triticum urartu* | 0 | 0.07±0 | 0 | 0.03±0 | 0 | 0 | 0 | 0.02±0.01 |
| *GCA-900066225* | 0.03±0 | 0 | 0 | 0 | 0.04±0.01 | 0.01±0.01 | 0.02±0 | 0.01±0.01 |
| uncultured organism *Lachnospiraceae* | 0 | 0 | 0 | 0 | 0 | 0 | 0.1±0 | 0 |
| uncultured *Caulobacteraceae* | 0.01±0 | 0.03±0 | 0 | 0 | 0.04±0 | 0 | 0.02±0 | 0.01±0.01 |
| *Ambiguous_taxa* | 0 | 0.07±0 | 0.02±0 | 0 | 0 | 0 | 0 | 0.02±0.01 |
| *Enhydrobacter* | 0 | 0.05±0 | 0 | 0.05±0 | 0 | 0 | 0 | 0.02±0 |
| *Shewanella* | 0 | 0 | 0 | 0.1±0 | 0 | 0 | 0 | 0.02±0 |
| *Ruminococcus 2* | 0.06±0 | 0 | 0.03±0 | 0 | 0 | 0 | 0 | 0.02±0.01 |
| *Brevundimonas* | 0 | 0.05±0 | 0 | 0.05±0 | 0 | 0 | 0 | 0.02±0 |
| *Eubacterium hallii group* | 0 | 0.09±0.01 | 0 | 0 | 0 | 0 | 0 | 0.02±0 |
| *Campylobacter* | 0.09±0.06 | 0 | 0 | 0 | 0 | 0 | 0 | 0.01±0 |
| *Anaerofilum* | 0.02±0 | 0.03±0 | 0 | 0 | 0.02±0 | 0.01±0 | 0 | 0.01±0 |
| *Aegilops tauschii* | 0 | 0 | 0 | 0.08±0 | 0 | 0 | 0 | 0.01±0 |
| *Family XIII UCG-001* | 0.07±0.02 | 0 | 0 | 0 | 0 | 0 | 0.01±0 | 0.01±0.02 |
| *gut metagenome* | 0 | 0 | 0 | 0 | 0.01±0 | 0 | 0.07±0 | 0 |
| *Peptococcus* | 0 | 0.05±0 | 0 | 0 | 0.03±0 | 0 | 0 | 0.01±0.01 |
| *Adlercreutzia* | 0.01±0 | 0.06±0.01 | 0 | 0 | 0 | 0 | 0 | 0.01±0.02 |
| *Bordetella* | 0.01±0 | 0.02±0 | 0.02±0 | 0.03±0 | 0 | 0 | 0 | 0.01±0 |
| *Modestobacter* | 0 | 0.03±0 | 0.03±0 | 0.01±0 | 0 | 0 | 0 | 0.01±0.01 |
| *Dorea* | 0 | 0.04±0 | 0.03±0 | 0 | 0 | 0 | 0 | 0.01±0.01 |
| *Bacillus* | 0.07±0 | 0 | 0 | 0 | 0 | 0 | 0 | 0.01±0 |
| *Luteibacter* | 0 | 0 | 0 | 0.07±0 | 0 | 0 | 0 | 0.01±0 |
| *Butyricimonas* | 0 | 0 | 0 | 0.06±0 | 0 | 0 | 0 | 0.01±0 |
| uncultured bacterium *Gastranaerophilales* | 0 | 0 | 0 | 0 | 0 | 0.01±0 | 0.05±0 | 0 |
| <0.01 | 0.42±0.12 | 0.62±0.21 | 0.24±0.2 | 0.23±0.17 | 0.24±0.08 | 0.09±0.03 | 0.21±0.11 | 0.31±0.08 |

**Table S8.** Overview of metagenomics sequencing results for each sample. Numerical numbers 1~3 indicate mouse numbers used in this experiment.

| Sample | Observed_otus | Shannon | Faith_pd | Evenness |
| --- | --- | --- | --- | --- |
| 1_Stomach | 236 | 5.03 | 32.82 | 0.64 |
| 1_Duodenum | 184 | 5.84 | 22.80 | 0.78 |
| 1_Jejunum | 144 | 5.86 | 24.99 | 0.82 |
| 1_Ileum | 171 | 5.47 | 33.00 | 0.74 |
| 1_Cecum | 769 | 8.07 | 73.43 | 0.84 |
| 1_Colon | 823 | 8.22 | 86.17 | 0.85 |
| 1_Feces | 645 | 7.93 | 60.92 | 0.85 |
| 2_Stomach | 927 | 8.36 | 78.70 | 0.85 |
| 2_Duodenum | 527 | 7.61 | 47.33 | 0.84 |
| 2_Jejunum | 323 | 6.61 | 26.42 | 0.79 |
| 2_Ileum | 392 | 7.14 | 30.59 | 0.83 |
| 2_Cecum | 788 | 8.13 | 67.61 | 0.84 |
| 2_Colon | 640 | 7.90 | 57.54 | 0.85 |
| 2_Feces | 641 | 7.85 | 55.14 | 0.84 |
| 3_Stomach | 230 | 6.87 | 45.17 | 0.88 |
| 3_Duodenum | 361 | 7.59 | 59.29 | 0.89 |
| 3_Jejunum | 366 | 6.91 | 31.49 | 0.81 |
| 3_Ileum | 368 | 6.71 | 32.68 | 0.79 |
| 3_Cecum | 911 | 8.41 | 78.44 | 0.86 |
| 3_Colon | 931 | 8.35 | 79.49 | 0.85 |
| 3_Feces | 364 | 7.17 | 42.75 | 0.84 |
